# Unveiling Glial Heterogeneity in the Inner Retina: Non-Visual Photic Spectrum Responses and Calcium Dynamics in Müller Glial Cells vs. Neurons

**DOI:** 10.3390/ijms27198879

**Published:** 2026-10-06

**Authors:** Maximiliano N. Rios, Natalia A. Marchese, Mario E. Guido

**Affiliations:** 1 CIQUIBIC-CONICET, Facultad de Ciencias Químicas, Universidad Nacional de Córdoba, Córdoba 5000, Argentina; natalia.marchese@unc.edu.ar; 2 Departamento de Química Biológica “Ranwel Caputto”, Facultad de Ciencias Químicas, Universidad Nacional de Córdoba, Córdoba 5000, Argentina

**Keywords:** Müller glial cells, calcium dynamics, retinal cells, light responses

## Abstract

Traditional calcium (Ca^2+^) imaging analysis often overlooks the non-stationary temporal complexity inherent in retinal circuits, reducing dynamic signaling traces to static summary descriptors. To resolve these dynamic signatures, we implemented a reproducible analytical framework combining Continuous Wavelet Transform (CWT) time-frequency decomposition with Sinc baseline filtering and Ward’s agglomerative hierarchical clustering, applied directly to standardized high-dimensional feature spaces, reserving Uniform Manifold Approximation and Projection (UMAP) strictly for low-dimensional visualization. This pipeline was deployed across three independent experimental models under blue light stimulation: mixed primary embryonic chicken retinal neurons, primary chicken Müller glial cells (MGCs), and the human MIO-M1 Müller cell line. Functional clustering resolved retinal neurons into three distinct kinetic subpopulations (k = 3), characterized by a kinetically stable core maintaining mid-frequency oscillations (Cluster 0), a cohort undergoing sharp post-stimulus period contraction with late amplitude surges (Cluster 1), and a low-frequency cohort with persistent period elongation (Cluster 2). Chicken MGCs partitioned into two operational clusters (k = 2), contrasting a period-stable, amplitude-decaying subpopulation (Cluster 0) with a dynamic subpopulation exhibiting light-evoked kinetic acceleration (Cluster 1). Similarly, human MIO-M1 glia separated into two major kinetic clusters (k = 2), distinguishing a low-amplitude, longer-period cohort from a high-amplitude, sustained subpopulation operating within an 8–80 s dynamic period band. Restricting phase-coupling and circular statistical analyses strictly to simultaneous Field-of-View (FOV) recordings revealed distinct stimulus-entrained network behaviors: primary avian MGCs maintain a decentralized, weakly coupled regime (“mosaic of autonomy”) that processes light across independent temporal windows, whereas human MIO-M1 glia and embryonic neuronal assemblies exhibit significant stimulus-evoked phase realignment and relative response latency sequencing. These findings demonstrate that inner retinal functional identity is fundamentally rhythmic and cluster-dependent, establishing a quantitative benchmark for decoding multi-scale Ca^2+^ signaling in healthy retinal preparations.

## 1. Introduction

The vertebrate retina is a highly specialized, laminated structure of the central nervous system capable of processing complex visual and non-visual photic information [1]. This vertical circuit is organized into three distinct cellular layers: the Outer Nuclear Layer (ONL), containing classical rod and cone photoreceptors; the Inner Nuclear Layer (INL), comprising interneurons (bipolar, horizontal, and amacrine cells) alongside the cell bodies of radial Müller glial cells (MGCs); and the Ganglion Cell Layer (GCL), populated by retinal ganglion cells (RGCs) and displaced amacrine cells [1]. For decades, retinal phototransduction was assumed to follow a strict hierarchical flow initiated exclusively by rod and cone hyperpolarization in the ONL. However, contemporary neurobiology has fundamentally rewritten this paradigm by establishing that the inner retina possesses autonomous light-sensing capabilities by non-visual opsins expression [2]. In the vertebrate inner retina, light detection within the blue and ultraviolet spectrum is governed by an integrated network of specialized photodetectors as melanopsin (Opn4), encephalopsin (Opn3) and neuropsin (Opn5) [1,3,4,5].

In both intrinsically photosensitive RGCs (ipRGCs) and developing horizontal cells, light-driven activation of Opn4 initiates a depolarizing signaling cascade coupled to a Gq/PLC/IP3 pathway [6,7,8,9]. This biochemical sequence triggers intracellular Ca^2+^ mobilization and the opening of membrane transient receptor potential canonical (TRPC) channels [6,8,10]. Beyond this rhabdomeric-type scheme, certain mammalian ipRGC subtypes also utilize ciliary-like mechanisms mediated by cyclic nucleotide-gated channels [11]. The coexistence of these parallel pathways underscores a profound deep operational heterogeneity in inner retinal photodetectors [1,12].

The structural and cellular complexity of the inner retinal non-visual photodetector matrix is significantly amplified by the complementary, heterochronic expression of Opn3 [13,14,15] and Opn5 [5,16]. While Opn3 functions primarily within the blue range of the visible spectrum [13,17], Opn5 acts as an evolutionarily conserved, ultraviolet (UV)-sensitive bistable photopigment coupled to Gi/o-protein signaling pathways [17,18]. In mammalian and avian retinas, both opsins exhibit an overlapping topographical distribution, mapping extensively throughout the GCL, the INL, and plexiform plexuses from early embryogenesis (E7–E10) into maturity [14,18,19]. Crucially, this non-visual opsin framework is not confined to neuronal lineages; it extends directly to glial cells. Primary cultures of avian MGC and the immortalized human MGC line MIO-M1 natively co-express Opn3 and Opn5 proteins [14,20]. In these macroglial systems, photic activation of Opn3 and Opn5 induces multi-scale biochemical shifts driven by de novo protein synthesis, downstream modulation of cyclic adenosine monophosphate (cAMP), and G-protein-mediated release of intracellular Ca^2+^ from internal endoplasmic reticulum stores [14,21]. Rather than driving uniform excitatory responses, the parallel operation of these non-visual opsins promotes long-lasting, highly heterogeneous intracellular Ca^2+^ dynamics. This specialized molecular arrangement empowers macroglial populations to generate multifaceted metabolic and kinetic states, structurally separating their temporal signaling languages from classical, fast neuronal circuits.

In this context, retinal neurons are characterized by fast variations in Ca^2+^ activity, operating on rapid timescales [22]. Conversely, Müller glial cells (MGCs) operate within a slow dynamic regime, generating complex, non-linear Ca^2+^ signals that evolve over timescales ranging from seconds to minutes. A core challenge in experimental characterization is the strong phenotypic heterogeneity inherent in these Ca^2+^ behaviors [20,22,23]. In neuronal and MGCs primary cultures, phenotypic heterogeneity and light environmental variations manifest as complex shifts in Ca^2+^ trace morphology rather than uniform changes in mean amplitude. For this reason, improved culture clusterization can be achieved following kinetic parameters such as oscillation duration, frequency, and the fraction of active cells triggering independent events, rather than relying on a single summary value [24,25].

To resolve the non-stationary complexities of inner retinal Ca^2+^ dynamics, an analytical framework capable of extracting time-varying features is essential. Biological Ca^2+^ signaling is inherently non-stationary; live-cell imaging recordings frequently present decaying amplitude envelopes, photobleaching artifacts, and non-linear baseline shifts that mathematically confound traditional frequency-domain operations, such as the Fast Fourier Transform (FFT) [26]. Classical algorithms routinely collapse multi-scale temporal recordings into static mean values, effectively masking critical, localized signaling alterations. Conversely, the continuous wavelet transform (CWT) framework provides exceptional time-frequency localization tailored for dynamic biological time-series [26,27]. By mapping individual cellular trajectories, this wavelet-based approach isolates high-resolution features of instantaneous amplitude, period, and phase. When coupled with downstream unsupervised machine learning, the extraction of these time-resolved wavelet ridges yields a robust analytical pipeline to quantify cellular functional heterogeneity and map the underlying topologic logic of neuro-glial networks.

In this study, we implemented a continuous time-frequency analytical framework using the computational platform pyBOAT to systematically characterize non-stationary inner retina Ca^2+^ dynamics evoked by photic stimulation recorded via confocal laser scanning microscopy. By applying Ward’s agglomerative hierarchical clustering directly to standardized high-dimensional feature vectors extracted via Continuous Wavelet Transform (CWT) analysis coupled with ideal Sinc filtering and model-tailored period boundaries we achieved an objective mathematical classification of distinct functional sub-populations. Rather than drawing direct cross-model statistical comparisons or speculative evolutionary claims across heterogeneous preparations, we leveraged three independent biological systems mixed primary embryonic chicken retinal neurons, primary chicken Müller glial cells (MGCs), and the human derived MIO-M1 cell line to demonstrate the versatility of this time resolved pipeline. Furthermore, by evaluating instantaneous phase-coupling dynamics strictly within individual Fields of View (FOVs), we mapped local network synchronization behaviors while avoiding over-interpretations of causal drive or directed control. Our findings reveal divergent light-evoked kinetic regimes, contrasting rapid period resetting in developing neurons with a decentralized ‘mosaic of autonomy’ in primary avian macroglia and stimulus-entrained phase coherence in human glia, thereby establishing a rigorous methodological standard for decoding complex intracellular Ca^2+^ trajectories in retinal assemblies.

## 2. Results

### 2.1. Population-Level Kinetic Profiling of Mixed Primary Embryonic Retinal Neurons

Agglomerative hierarchical clustering applied directly to the standardized high-dimensional feature space resolved the functional landscape of mixed primary embryonic chicken retinal cells (*N* = 352) into three distinct kinetic subpopulations (Figure 1A). Uniform Manifold Approximation and Projection (UMAP) embedding is utilized exclusively to visualize this two-dimensional spatial distribution. The numerical distribution identifies Cluster 0 as the predominant subpopulation (45.5%), followed by Cluster 2 (29.0%) and Cluster 1 (25.6%) (Figure 1B). The selection of k = 3 as the optimal functional partition was determined mathematically by a pronounced local maximum in the Calinski-Harabasz Index (peak value ~84.5; Figure S1C). Although higher partition sizes (k ≥ 4) yielded marginal increases in the Silhouette Score and lower Davies-Bouldin indices, selecting k > 3 induced severe population over-fragmentation, leaving insufficient single-cell counts per cluster for statistically robust downstream per-well/experimental unit analyses. Random Forest classification confirmed that subpopulation partitioning is primarily driven by early and late temporal kinetics: Wavelet Period Mean Early (Gini impurity decrease = 0.221), Wavelet Period Mean Late (0.213), and Amplitude Trajectory Delta (0.170) emerged as the top discriminatory features (Figure S1B). The integration of mean detrended intracellular Ca^2+^ fluorescence traces (ΔF/F0, arbitrary units) (Figure 1C), continuous instantaneous period curves (Figure 1D), time-resolved wavelet amplitude trajectories (Figure S1D) and single-cell spectral scalograms (Figure S1E) provides a descriptive quantitative profile of light-evoked, multi-phasic Ca^2+^ dynamics across the neuronal pool. Inter-cluster pairwise comparisons of these functional features formally characterize their kinetic identities (Figure 1E). Global inter-cluster evaluation using the omnibus Kruskal–Wallis test revealed highly significant differences among the three clusters across all primary metrics: Wavelet Period Mean Early (H = 172.97, *p* = 2.76 × 10^−38^), Wavelet Period Mean Late (H = 160.82, *p* = 1.20 × 10^−35^), and Amplitude Trajectory Delta (H = 164.64, *p* = 1.78 × 10^−36^) (Figure 1E).

- Cluster 0: Representing 45.5% of the cellular population, this cohort exhibits high oscillatory stability. It maintains a low instantaneous period trajectory constrained around ~30–36 s across the valid time window located outside the Cone of Influence (COI) (Median T_early = 29.55 s, IQR: 19.16–37.99 s; T_late = 35.82 s, IQR: 24.43–46.56 s; Figure 1E). Within-group comparison revealed a slight but significant period increase between the early and late phases (Wilcoxon signed-rank test, *p* = 7.74 × 10^−5^, Rosenthal effect size r = 0.320). In the time domain, its average wavelet amplitude profile displays a modest elevation following blue light stimulation (T = 60 s), rising from a ~0.35 a.u. baseline to ~0.40–0.42 a.u., before gradually returning to baseline levels during the late recovery window (Figure S1D). This kinetically balanced response is reflected by an Amplitude Trajectory Delta near zero (median = −0.026, IQR: −0.103 to 0.027; Figure 1E) and a continuous, stable mid-frequency band in single-cell scalograms outside COI artifact boundaries (Figure S1E).
- Cluster 1: Comprising 25.6% of the cellular population (*N* = 90), Cluster 1 undergoes a profound kinetic transition following light exposure. It displays a significantly elevated early period (Median T_early = 48.34 s, IQR: 40.45–59.31 s) that experiences a sharp, highly significant contraction during the late recovery phase (Median T_late = 24.04 s, IQR: 15.17–33.95 s; within-group Wilcoxon test, *p* = 6.36 × 10^−15^, with a strong effect size r = 0.861; Figure 1E), evaluated within the distortion-free COI window. In pairwise post-hoc comparisons with Benjamini–Hochberg FDR correction, the late period of Cluster 1 was significantly shorter than that of Cluster 0 (*p_adj_* = 1.34 × 10^−7^, r = 0.338) and Cluster 2 (*p_adj_* = 1.50 × 10^−30^, r = 0.838). Correspondingly, while its average amplitude remains moderate immediately post-stimulus (~0.28 a.u.), it exhibits a prominent, delayed surge that peaks (~0.40 a.u.) exclusively during the late recovery segment (Figure S1D). This descriptive profile is characterized by a strongly positive Amplitude Trajectory Delta (median = 0.127, IQR: 0.077–0.199; *p_adj_* = 7.07 × 10^−31^ vs. Cluster 0, r = 0.737; Figure 1E) and late spectral power intensification in continuous wavelet transform (CWT) scalograms outside COI edge-truncation zones (Figure S1E).
- Cluster 2: Accounting for 29.0% of the cells, Cluster 2 acts as a low-frequency oscillatory cohort defined by persistent period elongation. Its continuous period curve remains elevated and horizontal across both post-stimulus segments within the COI distortion-free window (Median T_early = 61.00 s, IQR: 54.43–69.77 s; T_late = 61.02 s, IQR: 48.45–69.49 s; Figure 1E). The within-group Wilcoxon test confirmed the absence of significant changes between the early and late periods (*p* = 0.394, r = 0.085), demonstrating an immediate and sustained kinetic deceleration relative to Clusters 0 and 1 (*p_adj_* < 10^−8^ across all pairwise comparisons; Figure 1E). Its time-domain amplitude profile exhibits an acute, transient spike (~0.35 a.u.) immediately upon photic onset (T = 60 s), followed by stabilization at a low, steady regime (~0.30 a.u.; Figure S1D). This trajectory yields a flat Amplitude Trajectory Delta (median = 0.004, IQR: −0.030 to 0.033; Figure 1E) and high spectral power concentrated within high-period (~60 s) in single-cell scalograms outside boundary COI regions (Figure S1E).

#### Field-of-View Network Topology and Light-Evoked Inter-Cluster Dynamic Phase

CouplingTo evaluate network functional interactions and avoid the incorrect assumption of physical coupling between cells originating from independent preparations, intercellular phase synchronization and coherence analysis was strictly restricted to cells recorded simultaneously within the same field of view (FOV/well).

Within this spatially and temporally coupled framework, the instantaneous phase (Φ(t)) extracted via the CWT in pyBOAT was used to quantify the PLV, or mean vector length R) and infer the relative response latency sequences among the previously defined functional subpopulations.

Intragroup synchronization (R(t)) and boundary masking in the safe analytical WindowBoundary regions at the beginning and end of the recording interval fall within the COI and are subject to spectral edge-truncation artifacts.Consequently, phase coherence measurements within these COI affected intervals including the early pre-stimulus baseline cannot be considered valid, and no physiological inferences (such as baseline stability or pre-to-post stimulus state shifts) were drawn from them. All intragroup coherence dynamics R(t) were evaluated strictly within the safe, COI-free analytical window.

In a representative FOV (well 3, *N* = 129 total cells: Cluster 0, *n* = 58; Cluster 1, *n* = 31; Cluster 2, *n* = 40), the temporal trajectory of the internal coherence parameter R(t) within the valid window revealed distinct post-stimulus profiles (Figure 2A):

- Cluster 0: Within the valid post-stimulus timeframe, Cluster 0 displayed a moderate, oscillating internal coherence regime (R(t) approx 0.3–0.5), showing a transient elevation following photic stimulation before stabilizing during the late recovery phase.
- Cluster 1: Within the safe analytical window, Cluster 1 exhibited low initial post-stimulus coherence (R(t) < 0.2), followed by a marked progressive rise during the late post-stimulus window, reaching a peak internal coherence (R(t) approx 0.5–0.6) that coincided with its late kinetic acceleration.
- Cluster 2: Maintained sustained, low-fluctuation internal coherence (R(t) approx 0.4–0.5) throughout the entire valid COI-free analytical window. Inter-Cluster Phase Coupling and Circular Statistics (Figure 2B) To assess whether photic stimulation induces modulations in phase alignment between cluster pairs within the valid analytical timeframe, a two-stage circular statistics framework was applied strictly across the safe post-stimulus early and late windows: Rayleigh Test for Circular Uniformity: To determine whether instantaneous phase differences (ΔΦ = Φ_A_ − Φ_B_) exhibit a statistically significant non-uniform distribution (*p* < 0.05) with a preferential mean angle (Mean ΔΦ).

Two-Sample Circular Permutation Test (*N* = 2000 permutations) with Benjamini–Hochberg FDR Correction: To evaluate the statistical significance of the change in phase coupling strength (ΔR = R_late − R_early) between both valid post-stimulus windows.

In representative well 3 (Figure 2B, Supplementary Table S1), all three cluster pairs displayed highly significant non-uniform phase distributions in both valid post-stimulus segments (Rayleigh Test, *p* < 0.03 in all cases), confirming the presence of defined phase-locked states.

- C0–C2 Pair: Showed a statistically significant phase coupling modulation (ΔR = +0.239$, *p_raw_* = 5.00 × 10^−3^, *p_FDR_* = 0.011; Figure 2B, middle panel). In the early post-stimulus segment, the phase relationship reflected weak coupling (PLV R = 0.199, Rayleigh *p* = 0.029; Mean ΔΦ = −0.03 rad, −1.8°). During the late phase, the pair showed a marked reinforcement in phase coherence (R = 0.439, Rayleigh *p* = 1.26 × 10^−7^), accompanied by a shift in the mean direction to Mean ΔΦ = +0.87 rad (+50.1°), reflecting a transition toward a relative response latency where Cluster 0 leads Cluster 2 in phase.
- C0–C1 Pair: Exhibited a modest increase in phase coherence within the safe window (from R = 0.264, Mean ΔΦ = +0.99 rad (+56.9°) in the early phase to R = 0.388, Mean ΔΦ = +0.34 rad (+19.7°) in the late phase; ΔR = +0.125). However, this change did not reach significance following false discovery rate correction (*p_raw_* = 0.125, *p_FDR_* = 0.141; Figure 2B, left panel).
- **C1–C2 Pair:** Displayed a trend toward increased coupling strength (from R = 0.311, Mean ΔΦ = −0.21 rad (11.9°) to R = 0.460, Mean ΔΦ = +0.44 rad (+25.5°); ΔR = +0.149, *p_raw_* = 0.061), but was not statistically significant after FDR adjustment (*p_FDR_* = 0.078; Figure 2B, right panel).

Cross-FOV Variability and Network-Specific DynamicsComparative analysis extended across multiple independent fields of view (well 1, well 3, and well 4) within safe analytical windows demonstrated that inter-cluster phase coupling is dependent on the local network topology of each preparation (Supplementary Table S1).

### 2.2. Population-Level Kinetic Profiling of Primary Embryonic Retinal Müller Glial Cells (MGCs)

Agglomerative hierarchical clustering applied directly to the standardized high-dimensional feature space resolved the functional landscape of primary embryonic chicken Müller Glial Cells (MGCs) (*N* = 202) into two distinct kinetic subpopulations (Figure 3A, Figure S2A). UMAP embedding is utilized exclusively to visualize this two-dimensional spatial distribution (Figure 3A). The numerical distribution identifies Cluster 0 as the predominant subpopulation (56.4%), followed by Cluster 1 (43.6%) (Figure 3B). The selection of k = 2 as the optimal functional partition was determined mathematically by a pronounced peak in the Calinski-Harabasz Index (peak value = 83.5), a global minimum in the Davies-Bouldin Index (1.44), and a maximum in the Silhouette Score (0.26) (Figure S2C). Random Forest classification confirmed that subpopulation partitioning is primarily driven by post-stimulus trajectory and late oscillatory period metrics: Amplitude Trajectory Delta (Gini impurity decrease = 0.275), Wavelet Period Mean Late (0.271), and Period Trajectory Delta (0.171) emerged as the top discriminatory features (Figure S2B). The integration of mean detrended intracellular Ca^2+^ fluorescence traces (ΔF/F0, arbitrary units) (Figure 3C), continuous instantaneous period curves (Figure 3D), time-resolved wavelet amplitude trajectories (Figure S2D), and representative single-cell spectral scalograms from well_3 (Figure S2E) provides a descriptive quantitative profile of light-evoked, multi-phasic Ca^2+^ dynamics across the MGC pool. Inter-cluster pairwise comparisons of these functional features formally characterize their kinetic identities (Figure 3E). Global inter-cluster evaluation using the omnibus Kruskal–Wallis test revealed highly significant differences between the two clusters across all primary metrics: Amplitude Trajectory Delta (H = 126.97, *p* = 1.88 × 10^−29^), Wavelet Period Mean Late (H = 108.70, *p* = 1.89 × 10^−25^), and Period Trajectory Delta (H = 96.99, *p* = 6.95 × 10^−23^) (Figure 3E).

- Cluster 0: This cellular population exhibits high oscillatory period stability. It maintains a sustained dominant period trajectory constrained around 46–49 s across the valid time window located outside the COI (Mean T_early = 48.96 s, IQR: 44.18–53.73 s; T_late = 46.19 s}, IQR: 34.29–58.87 s; Figure 3D,E). Within-group comparison revealed no statistically significant shift in period between the early and late phases (Wilcoxon signed-rank test, *p* = 0.153, Rosenthal effect size r = 0.142). In the time domain, its average wavelet amplitude profile displays a progressive, sustained attenuation following blue light stimulation (T = 45 s), declining from a 0.50 a.u. baseline to 0.33–0.35 a.u. during the late recovery window (Figure S2D). This kinetically decaying response is reflected by a significantly negative Amplitude Trajectory Delta (median = −0.104, IQR: −0.175 to −0.059; Figure 3E) and a flat Period Trajectory Delta centered near zero (IQR: −7.19 to 3.00 s; Figure 3E). Single-cell representative scalograms from well_3 confirm continuous, stable spectral power concentrated within high-period bands (45–50 s) outside COI artifact boundaries (Figure S2E).
- Cluster 1: This cellular population undergoes a profound kinetic transition following light exposure. It displays an elevated early period (Median T_early = 45.62 s, IQR: 36.42–54.82 s) that experiences a sharp, highly significant contraction during the late recovery phase (Median T_late = 17.02 s, IQR: 12.29–23.65 s; within-group Wilcoxon test, *p* = 3.47 × 10^−15^, with a strong effect size r = 0.864; Figure 3D,E), evaluated within the distortion-free COI window. In pairwise post-hoc comparisons with Benjamini–Hochberg FDR correction, the late period of Cluster 1 was significantly shorter than that of Cluster 0 (*p_adj_* = 1.92 × 10^−25^, r = 0.766; Figure 3E). Correspondingly, while its average amplitude remains moderate immediately post-stimulus (0.33 a.u.), it exhibits a prominent, delayed surge that peaks (0.45 a.u.) exclusively during the late recovery segment (Figure S2D). This descriptive profile is characterized by a strongly positive Amplitude Trajectory Delta (median = 0.089, IQR: 0.029–0.159; *p_adj_* = 1.91 × 10^−29^ vs. Cluster 0, r = 0.793; Figure 3E) and a strongly negative Period Trajectory Delta (median = −24.79 s, IQR: −31.44 to −17.27 s; *p_adj_* = 7.05 × 10^−23^ vs. Cluster 0, r = 0.726; Figure 3E). Continuous wavelet transform (CWT) scalograms demonstrate a distinct spectral shift, with power intensifying in short-period bands (15–20 s) during late recovery outside COI (Figure S2E)

#### Intra-Cluster Synchronization and Inter-Cluster Phase Coupling in Chicken MGCs

In a representative FOV (well 3, *N* = 61 total cells: Cluster 0, *n* = 36; Cluster 1, *n* = 25), evaluation of internal coherence R(t) was strictly constrained to valid analytical windows, explicitly excluding edge-artifact-prone regions invalidated by the Cone of Influence (COI) boundary (Figure 4A). Within this safe post-stimulus timeframe delineated into early and late post-stimulus segments by the boundary glial subpopulations exhibited distinct intrinsic synchronization profiles (Figure 4A):

- Cluster 0: Within the safe analytical window, Cluster 0 displayed a moderate, gradually decaying internal coherence regime (R(t) approx 0.5–0.3. It maintained elevated baseline coherence prior to photic stimulation (T = 45 s), followed by a continuous, progressive decline throughout the post-stimulus phase and late recovery window (Figure 4A).
- Cluster 1: Within the valid COI-free region, Cluster 1 exhibited lower and more transient internal coherence (R(t) (approx 0.1–0.4), marked by transient coherence oscillations immediately post-stimulus (T = 45 s) before remaining in a low-coherence state across both early and late analytical segments (Figure 4A).

Inter-Cluster Phase Coupling and Circular Statistics (Figure 4B) in representative well 3 (*N* = 59 cells analyzed for the C0–C1 pair; Figure 4B, Supplementary Table S2), inter-cluster phase coupling exhibited the following dynamic behavior:

C0–C1 Pair: In the early post-stimulus segment, the pair demonstrated a highly significant non-uniform phase distribution PLV R = 0.416, Rayleigh test *p* = 2.51 × 10^−5^) centered at a mean direction of ΔΦ = +0.45 rad (+25.6°), reflecting a phase-locked state where Cluster 0 slightly leads Cluster 1 in phase. During the late post-stimulus segment, phase alignment dissipated into a uniform distribution (PLV R = 0.278, Rayleigh test *p* = 0.124 (ns) with a shifted mean angle (Mean ΔΦ = +1.29 rad, +74.2°). Although coupling strength decreased (Delta R = −0.138), the two-sample circular permutation test confirmed that this shift did not reach statistical significance following false discovery rate adjustment (*p_raw_* = 0.126, *p_FDR_* = 0.380; Figure 4B). Cross-FOV Variability and Network-Specific DynamicsComparative analysis extended across multiple independent fields of view (well 1, well 2, and well 3) within safe analytical windows confirmed that MGC inter-cluster phase coupling remains in an uncoupled or weakly coupled regime across local glial networks (Supplementary Table S2).

### 2.3. Population-Level Kinetic Profiling of Human Müller Glial Cells (MIO-M1)

Agglomerative hierarchical clustering applied directly to the standardized high-dimensional feature space resolved the functional landscape of human Müller glial cells (MIO-M1 line, *N* = 240) into two distinct kinetic subpopulations (Figure 5A and Figure S3A). UMAP embedding is utilized exclusively to visualize this two-dimensional spatial distribution (Figure 5A). The numerical distribution identifies Cluster 0 as the predominant subpopulation (51.7%), closely followed by Cluster 1 (48.3%) (Figure 5B). The selection of k = 2 as the optimal functional partition was determined mathematically by a pronounced local maximum in the Calinski-Harabasz Index (peak value 57.6; Figure S3C) and the hierarchical dendrogram tree-cut threshold (Figure S3A). Although the k = 3 partition yielded a marginal increase in the Silhouette Score (0.190 vs. 0.185) and a lower Davies-Bouldin Index (1.49 vs. 1.85), selecting k > 2 induced severe population over-fragmentation, leaving insufficient single-cell sample sizes per cluster for statistically robust downstream experimental unit analyses. Random Forest classification confirmed that subpopulation partitioning is primarily driven by early amplitude, early period, and late signal-to-noise ratio modulation: Wavelet Amplitude Mean Early (Gini impurity decrease = 0.165), Wavelet Period Mean Early (0.159), and SNR Late vs. Basal log_2 FC (0.135) emerged as the top discriminatory feature (Figure S3B). The integration of mean detrended intracellular Ca^2+^ fluorescence traces (ΔF/F0, arbitrary units) (Figure 5C), continuous instantaneous period curves (Figure 5D), time-resolved wavelet amplitude trajectories (Figure S3D), and single-cell spectral scalograms (Figure S3E) provides a descriptive quantitative profile of light-evoked, multi-phasic Ca^2+^ dynamics across the MIO-M1 glial pool. Inter-cluster pairwise comparisons of these functional features formally characterize their kinetic identities (Figure 5E). Global inter-cluster evaluation using the omnibus Kruskal–Wallis test revealed highly significant differences between the two clusters across all primary metrics: Wavelet Amplitude Mean Early (H = 51.631, *p* = 6.70 × 10^−13^), Wavelet Period Mean Early (H = 38.280, *p* = 6.13 × 10^−10^), and SNR Late vs. Basal log_2FC (H = 33.052, *p* = 8.97 × 10^−9^) (Figure 5E).

- Cluster 0: This cellular population is characterized by a low initial response amplitude combined with an elongated early oscillatory period. It maintains an elevated early period trajectory centered around a median of 65.47 s (IQR: 54.89–74.03 s; Figure 5D,E) within the valid analytical time window located outside the COI. Following blue light stimulation (T = 60 s), it undergoes a statistically significant period contraction during the late recovery phase (Mean T_late = 54.83 s, IQR: 32.00 s; within-group Wilcoxon signed-rank test, *p* = 1.92 × 10^−5^, Rosenthal effect size r = 0.384; Figure 5D). In the time domain, its average wavelet amplitude profile remains low during the early post-stimulus phase (Median = 0.249 a.u., IQR: 0.199–0.303 a.u.; Figure 5E), showing a modest post-stimulus rise up to 0.30 a.u. before decaying back toward 0.20 a.u. in the late window (Figure S3D). Furthermore, it displays a negative log-fold ratio in SNR during late recovery relative to baseline (Mean SNR log_2 FC = −0.233, IQR: −0.427 to −0.050; Figure 5E). Single-cell representatives from well_2 scalograms confirm continuous spectral power concentrated in higher-period bands (55–70) outside COI artifact boundaries (Figure S3E).
- **Cluster 1:** This cluster acts as a high-amplitude, shorter-period subpopulation with superior signal stability. Within the distortion-free COI window, it exhibits a significantly shorter early oscillatory period (Mean T_early = 51.27 s, IQR: 42.52–63.57 s; Figure 5D,E) compared to Cluster 0 (*p_adj_* = 6.16 × 10^−10^, r = 0.399; Figure 5E). Within-group temporal evaluation demonstrated a modest yet significant period contraction between the early and late segments (Mean T_late = 45.75 s, IQR: 22.1 s; within-group Wilcoxon test, *p* = 8.59 × 10^−3^, r = 0.244; Figure 5D). Correspondingly, its average amplitude displays a rapid and sustained elevation following photic onset (T = 60 s), reaching a high early mean amplitude (Median = 0.328 a.u., IQR: 0.279–0.383 a.u.; *p_adj_* = 6.74 × 10^−13^ vs. Cluster 0, r = 0.464; Figure 5E) that peaks at 0.35 a.u.and remains sustained (0.32 a.u.) throughout the late phase (Figure S3D). This persistent kinetic response is reflected in a stable late vs. basal SNR log-fold change near baseline (Median SNR log_2FC = −0.050, IQR: −0.192 to 0.117; *p_adj_* = 9.02 × 10^−9^ vs. Cluster 0, r = 0.371; Figure 5E) and spectral power focused within faster oscillation bands (40–50 s) outside COI (Figure S3E).

#### Field-of-View (FOV) Level Analysis: Intra-Cluster Synchronization and Inter-Cluster Phase Coupling in Human MIO-M1 Müller Glia

In a representative FOV (well 2, *N* = 35 total cells: Cluster 0, *N* = 16; Cluster 1, *N* = 19), evaluation of internal coherence R(t) was strictly constrained to valid analytical windows, explicitly excluding edge-artifact-prone regions invalidated by the Cone of Influence (COI) boundary where time-frequency signal decomposition suffers from edge distortion (Figure 6A). Within this safe delineated into early and latempost-stimulus segments following photostimulation onset at T = 60 s, human MIO-M1 Müller glia displayed robust, cluster-specific internal synchronization profiles (Figure 6A):

- Cluster 0: Upon entering the valid post-COI window, initially this cluster displayed a low internal coherence regime (R(t) 0.10–0.20, followed by a progressive and continuous recruitment across the early post-stimulus segment, eventually consolidating into a highly coherent state R(t) 0.80–0.85 during the late recovery phase (Figure 6A).
- Cluster 1: Within the distortion-free analytical window, Cluster 1 exhibited remarkably sustained, elevated internal synchronization throughout the entire observation window (R(t) 0.60–0.80), maintaining strong cohort cohesion across both early and late post-stimulus segments (Figure 6A).

Inter-Cluster Phase Coupling and Circular Statistics (Figure 6B), in representative well 2 (*N* = 35 cells analyzed for the C0–C1 pair; Figure 6B, Supplementary Table S3), inter-cluster phase coupling exhibited a strong, stimulus-evoked phase locking shift: C0–C1 Pair: In the early post-stimulus segment, the pair demonstrated a highly significant non-uniform phase distribution PLV R = 0.560, Rayleigh test *p* = 7.30 × 10^−6^) centered at a mean direction of Mean ΔΦ = +1.49 rad (+85.2°), reflecting a phase-locked state where Cluster 0 leads Cluster 1 by approximately a quarter-cycle (90°). During the late post-stimulus segment, phase alignment further intensified and realigned into a highly synchronized, near-zero phase-locked state (PLV R = 0.770, Rayleigh test *p* = 1.14 × 10^−9^) with a shifted mean angle of Mean ΔΦ = −0.02 rad (−1.4°). This substantial increase in coupling strength (Delta R = +0.210) was confirmed to be statistically significant by the two-sample circular permutation test following FDR adjustment (*p_raw_* <0.001, *p_FDR_*< 0.001; Figure 6B).

Cross-FOV Variability and Network-Specific DynamicsComparative analysis extended across six independent fields of view (wells 1–6) within COI-free analytical windows revealed that human MIO-M1 glial networks possess localized capacity for dynamic phase coupling (Supplementary Table S3).

## 3. Discussion

### 3.1. Divergent Kinetic Trajectories: Rapid Neuronal Resetting vs. Glial Kinetic Transitions

Our quantitative profiling uncovers a fundamental divergence in the light-evoked kinetic regimes of embryonic retinal neurons and Müller glial cells under their respective photic stimulation regimes (T_stim_start = 60.0 s for neurons and MIO-M1; T_stim_start = 45.0 s for chicken MGCs). It is important to remark on that mixed primary embryonic retinal neurons as retinal cultures contain multiple types of neurons in the early developmental retina [28]. We evaluated across an evaluated period window of 8.0–80.0 s, partitioning into a three-cluster functional architecture (k = 3) (Figure 1A,B). The predominant subpopulation, Cluster 0 (45.5%), forms a kinetically stable core maintaining a narrow instantaneous period window (approx.. 30–36 s) across early post-stimulus phases, showing a tendency to increase in the late segment to 40–50 s (Figure 1D). In contrast, Cluster 1 (25.6%) undergoes a rapid, light-evoked kinetic transition characterized by an initial elevated period (Mean T_early = 48.34 s) that contracts sharply during late recovery (Mean T_late = 24.04 s), accompanied by late spectral amplitude intensification (Figure 1D,E). Cluster 2 (29.0%) operates as a low-frequency cohort marked by persistent periods (Median T_early = 61.00 s, T_late = 61.02) (Figure 1D,E).

Primary embryonic chicken MGCs, evaluated, exhibit a distinct two-cluster architecture (k = 2) dominated by slow-scale oscillatory trajectories. Cluster 0 (56.4%) maintains high period stability (approx 46–49 s) accompanied by progressive amplitude decay following photic onset (Figure 3D and Figure S2D). Conversely, Cluster 1 (43.6%) displays a marked post-stimulus kinetic acceleration, contracting its dominant period from 45.62 s early to 17.02 s late (Period Trajectory Delta = −24.79 s) alongside a delayed surge in wavelet amplitude (Figure 3D and Figure S2D).

In the human MIO-M1 cell line, functional partitioning similarly yields two major subpopulations. Cluster 0 (51.7%) exhibits an initial low-amplitude response with an early period of 65.47 s that contracts modestly to 54.83 s late (Figure 5D,E and Figure S3D). Cluster 1 (48.3%) represents a high-amplitude, sustained responder operating within faster period bands (Mean T_early = 51.27 s, T_late = 45.75 s) with highly stable post-stimulus signal-to-noise ratios (Figure 5D,E and Figure S3D). By setting a Sinc cutoff period of T_cutoff = 100.0 s prior to CWT execution, non-stationary baseline fluctuations exceeding 100.0 s were isolated as background drift, ensuring that extracted ridge trajectories accurately reflect genuine multi-cycle oscillatory rhythms within the evaluated 6.0–80.0 dynamic band.

### 3.2. Field-of-View Network Topology and Stimulus-Entrained Phase Dynamics

To evaluate intercellular phase relationships without assuming physical or functional connectivity between cells from separate preparations, phase coupling analyses were restricted strictly to cells recorded simultaneously within individual FOVs/wells. Instantaneous phase (Φ(t)) and mean vector length (R(t)) dynamics evaluated within the distortion-free analytical window (strictly excluding edge boundary regions defined by the COI) reveal distinct network synchronization behaviors across models.In representative neuronal FOVs, circular statistical evaluation (Rayleigh test for non-uniformity and two-sample circular permutation tests with FDR adjustment, see Section 4.4.5) demonstrates significant light-evoked phase alignment. Specifically, the C0–C2 neuronal cluster pair displays a significant post-stimulus shift in phase coupling strength (Delta R = +0.239), transitioning from weak early coupling (R = 0.199) to sustained late phase-locking (R = 0.439) with a preferential mean direction of ΔΦ = +0.87 rad (+50.1°) (Figure 2B, Supplementary Table S1). This directional mean indicates a relative response latency sequence where Cluster 0 leads Cluster 2 in phase (see Section 4.4.5 and Figure 2B, Supplementary Table S1). Rather than implying causal drive, this phase relationship reflects stimulus-entrained temporal response sequencing across distinct neuronal subpopulations.

In primary avian MGC networks, inter-cluster phase coupling operates within an uncoupled or weakly coupled regime. In representative FOVs, the C0–C1 glial pair exhibits initial transient phase locking post-stimulus (R = 0.416, Mean ΔΦ = +0.45 rad) that dissipates into a uniform phase distribution during late recovery (R = 0.278) (see Section 4.4.5, Figure 4B, and Supplementary Table S2). This decay in phase coherence confirms that primary glial cells do not function as a centrally synchronized syncytium during blue light processing, but instead maintain a decentralized “mosaic of autonomy” where individual clusters process photic information across independent, staggered temporal windows. Conversely, human MIO-M1 glial networks within individual FOVs display a high capacity for stimulus-evoked phase synchronization. Following photic onset, MIO-M1 clusters demonstrate a marked increase in phase coherence (Delta R = 0.210), realigning from an initial phase-lag state (Mean Delta ΔΦ = +1.49 rad) into a highly coherent, near-zero phase-locked regime (R = 0.770 Mean ΔΦ = −0.02 rad) (See Section 4.4.5, Figure 6B, Supplementary Table S3). This demonstrates that local human glial assemblies can recruit tightly synchronized oscillatory states under continuous light exposure.

### 3.3. Mechanistic Foundations and Experimental Model Considerations

The light-evoked intracellular Ca^2+^ dynamics observed in primary avian Müller glia build upon established non-visual phototransduction cascades. Avian MGCs natively express the non-visual opsins Opn3 and Opn5, which trigger G_q/o_ protein activation, phospholipase C (PLC) engagement, and subsequent inositol 1,4,5-trisphosphate (IP_3_-mediated Ca^2+^ release from internal endoplasmic reticulum stores [14,21,23]. This intrinsic pathway drives sustained, long-period Ca^2+^ oscillations that persist long after stimulus offset [29,30]. In human MIO-M1 glia, while native expression of opsin transcripts (including OPN1SW and OPN4) has been reported [20,31], the current study characterizes light-evoked kinetic profiles without direct genetic knockout or pharmacological receptor blockade. Therefore, these responses are defined strictly as light-evoked functional Ca^2+^ kinetics. Care must be taken when contextualizing findings across the three evaluated models. Primary chicken neurons, primary chicken MGCs, and immortalized human MIO-M1 cells represent distinct biological preparations that differ in species, developmental stage, cellular composition, fluorophore selection (Calcium Orange vs. Fluo-4), and experimental performance (see Section 4.3). Instead, each preparation serves as an independent biological validation demonstrating the versatility of the time-frequency computational pipeline across diverse experimental configurations. Future loss-of-function studies using RNA interference or specific phototransduction inhibitors will be required to definitively dissect the relative contributions of intrinsic opsin cascades versus extrinsic signaling mechanisms in human glia as we previously demonstrate in chicken MGCs [14,21].

And research combining continuous time-frequency analysis with targeted pharmacological manipulations such as thapsigargin for endoplasmic reticulum Ca^2+^-ATPase inhibition, will clarify the precise biochemical microdomains that drive cluster-specific kinetic transitions. Furthermore, extending single-FOV phase coupling maps to spatially resolved distance matrices will clarify how functional oscillatory synchronization scales with physical intercellular distance across the retinal tissue.

## 4. Materials and Methods

### 4.1. Primary Cultures of Chicken Müller Glial and Retinal Neuron Cells

Primary cultures of Müller glial and retina neuron cells were purified from neural retinas of day 8 embryonic (E8) chicks dissected in ice-cold Tyrode’s buffer without Ca^2+^ or Mg^2+^, containing 25 mM glucose, as previously described 13,106. In summary, the cells were treated with papain (P3125 Sigma-Aldrich (St. Louis, MO, USA)) for 25 min at 37 °C and with deoxyribonuclease I (18047-019 Invitrogen (Carlsbad, CA, USA)) and were rinsed with 10% fetal bovine serum (FBS) and half Dulbecco’s Modified Eagle Medium (DMEM). After dissociation, to perform MGCs culture the cells in suspension were seeded into 60 mm Petri dishes and cultured in DMEM supplemented with 10% for at least one week; the cultures were then passaged using trypsin into a 8-well Lab-Tek recording chamber (NuncTM, New York, NY, USA) and allowed to grow for another week in DMEM supplemented with 10% FBS. While preparing the neuron cells culture, the cell suspension were seeded in an 8-well Lab-Tek recording chamber containing “Super N” medium; DMEM supplemented with B27 (Life Technologies, Invitrogen(Carlsbad, CA, USA), GIBCO(Grand Island, NY, USA); dilution: 1/50 *v*/*v*) and 4 μM forskolin (Cayman, Ann Arbor, MI, USA,) for three days. The MGCs and neuron cultures were maintained at 37 °C under a constant flow of air and 5% CO_2_ in a humid atmosphere. For each experiment in primary cultures for neurons and MGCs, three chicken embryos were dissected, collecting the eyes in pairs. Treating these cultures as 1 sample per embryo, the total of recordings is the summary of independent experiments.

### 4.2. Human MIO-M1 Cell Culture

The human Müller cell line (Moorfields/Institute of Ophthalmology-Müller 1) MIO-M1, was generated and previously characterized by G. Astrid Limb lab [29] and kindly provided by Cecilia Sanchez. After receiving cells to generate the stocks a mycoplasma test using Hoesch was performed to avoid cell contamination (Image in Supplementary Materials). We use the cells between 5 to 10 passages for experiments. Cells were cultured in 24-well plates (Biologix (Camarillo, CA, USA)) on circular coverslips 12 mm in diameter, with 15,000 cells seeded per well, using modified DMEM supplemented with 10% FBS and penicillin/streptomycin, the cultures were maintained in 5% CO_2_ at 37 °C during 2 days for calcium recording. A total of 6 independent experiments were performed with MIO-M1 Cells.

### 4.3. Calcium Imaging by Fluorescence Microscopy

Primary cultures of MGCs and neurons were grown in an 8-well Lab-Tek recording chamber (NuncTM, Rochester, NY, USA) in DMEM + SFB 10% and Super N medium respectively, while de coverslips with growing MIO-M1 cells were mounted in a 12 mm 3D printed chamber designed by GOATLAB [32], an open source repository for research equipment. On the day of the experiment, MGCs and MIO-M1 were incubated with 0.1% of pluronic acid F-127 and the Ca^2+^ indicator dye Calcium Orange AM 5 μM (Invitrogen–Molecular Probes) and Fluo-4 AM was used for the neurons and MIO-M1 cultures, both in DMEM for 20 min at 37 °C, under darkness condition. The fluorescence imaging technique was performed by using the Ca^2+^ sensitive indicator calcium orange-AM excited at 543 nm and 495 nm for Fluo-4 AM with a laser coupled to a confocal microscope (Olympus FluoView-1000 (Tokyo, Japan)). The emitted fluorescence was captured every 2 s, using a PlanApo N 60 × Uplan SApo oil-immersion objective (NA: 1.42; Olympus). The 12 bit 4 × 4 binned fluorescence images for each photo were used to quantify fluorescence levels in the cells using ImageJ v1.54r; the mean fluorescence intensity in each cell was background-corrected by subtracting the mean fluorescence of an area with no cells (only cells showing positive fluorescence pre-stimuli were considered for the analysis) and motion correction using StackReg v2.0.0 plugin in FIJI. The mean intensity of individual cells was measured in each captured image series in circular ROIs (Region of Interest) per cell and saved to the ROI Manager. Changes in fluorescence levels were quantified as the ratio between each relative intensity level measured after a blue light stimulus of 68 μW/cm^2^ for 20 s (F) and the mean of intensities of serial pictures before stimulation (Fo), the same measurements were quantified in the background to correct F/Fo for each cell, as described in [8].

### 4.4. Computational Analysis and Functional Clustering

#### 4.4.1. Signal Processing and Time-Frequency DecompositionRaw

Intracellular Ca^2+^ fluorescence time-series (F) were processed using a custom, reproducible computational pipeline integrated with the pyBOAT framework (v1.1.1). To eliminate non-stationary low-frequency baseline drifts and photobleaching artifacts without introducing spectral leakage or high-frequency distortion, signals were detrended in the frequency domain using a sinc-filter (brick-wall filter) with model-specific cutoff periods (T_cutoff = 75.0–100.0 s). Time-frequency decomposition was executed via Continuous Wavelet Transform (CWT) using a complex Morlet wavelet basis, providing optimal joint time-frequency resolution for non-stationary biological oscillations. Wavelet power spectra were evaluated across model-tailored period ranges discretized into 100 to 150 linearly spaced period steps. Model-specific sampling intervals (Δt) were explicitly defined at 1.0 s for MIO-M1 cells and 2.0 s for Neurons and MGCs. Time-resolved rhythmic metrics including instantaneous period, phase, and envelope amplitude were extracted along the maximum power ridge of the wavelet scalogram. To eliminate background noise and spurious fluctuations, ridge extraction was regularized using a minimum power threshold (Power Threshold = 3.0), a maximum power scaling factor (Max Power Scale = 25.0), and a temporal envelope smoothing window (W_envelope = 25.0–30.0 s). To strictly prevent edge-effect distortions, all time-frequency metrics and downstream phase calculations were restricted to valid spectral regions outside the Cone of Influence (COI), defined by the e-folding time of the Morlet wavelet. Parameters are resumed in the Table 1:

#### 4.4.2. Temporal Segment Feature Engineering

Feature extraction was executed using a dedicated transformer module (PureWaveletSegmentedExtractor), which partitioned each single-cell time-series into three predefined experimental phases: Basal (T < onset stimulus), Early Post-Stimulus, and Late Post-Stimulus. For each temporal phase, comprehensive dynamic features were computed, including standard deviation, skewness, mean wavelet period (e.g., wavelet_period_mean_early), mean wavelet amplitude (e.g., wavelet_amplitude_mean_late), and Signal-to-Noise Ratio (SNR). Furthermore, temporal trajectory deltas (ΔPeriod and ΔAmplitude (late- early) and logarithmic fold-changes (log2FC) relative to baseline were calculated to quantify stimulus-evoked adaptation dynamics across functional stages.

#### 4.4.3. Data Preprocessing, Dimensionality Reduction, and Hierarchical Clustering

To eliminate experimental batch effects and field-of-view (FOV) variability across culture wells, extracted features were first subjected to intra-well Z-score normalization. Missing values were imputed using median estimation, followed by variance thresholding and collinearity pruning (Pearson correlation coefficient |r| > 0.85) to remove redundant metrics. To satisfy Gaussian distribution assumptions required for linear projection, a Yeo-Johnson power transformation was applied to the cleaned feature matrix. Dimensionality reduction was conducted via Principal Component Analysis (PCA), retaining orthogonal components that captured a pre-specified cumulative explained variance threshold (>85%). To avoid topological distance distortions inherently introduced by non-linear manifold embeddings, Agglomerative Hierarchical Clustering with Ward’s minimum variance linkage was applied directly to the PCA-transformed high-dimensional feature space. Uniform Manifold Approximation and Projection (UMAP) was employed exclusively as a post-hoc visualization tool to display the two-dimensional spatial distribution of the pre-identified clusters.

#### 4.4.4. Cluster Validation, Supervised Machine Learning, and Feature Discriminative Power

The optimal partition of functional clusters (k) was determined mathematically through multi-metric evaluation across a range of potential cluster numbers (k = 2–10). Clustering performance was assessed by maximizing the Calinski-Harabasz Index and Silhouette Score while minimizing the Davies-Bouldin Index, aligned with dendrogram tree-cut threshold analysis. To characterize cluster separability and identify key phenotypic drivers, a Random Forest (RF) classifier was trained on the unprojected feature matrix to predict cluster assignments. To prevent overfitting, regularized hyperparameter constraints were implemented (maximum tree depth = 5, minimum samples per leaf = 15). Feature importance was quantified using the Mean Decrease in Gini Impurity. Potential experimental bias across physical culture wells was formally tested using Chi-square (χ^2^) test of independence on cluster population distributions per FOV (Supplementary S4).

#### 4.4.5. COI-Filtered Time-Resolved Coherence and Circular Phase Coupling

The coordination within individual FOVs was evaluated by computing the instantaneous Phase Locking Value (PLV), R(t), across cells assigned to the same functional cluster, excluding temporal segments falling within the COI edge-artifact zones: R(t) = 1/N|∑j = 1N ei Φj(t)| where Φj(t) represents the instantaneous phase of cell j at time t, and N is the total number of cells within the cluster. Inter-cluster phase coupling between cluster pairs (C0–C1) was evaluated by calculating instantaneous phase differences (ΔΦ = ΦC0 − ΦC1) across COI-free early and late post-stimulus windows. Circular distribution concentration and mean phase angles (Mean ΔΦ) were assessed using the Rayleigh Test for Circular Uniformity. To test whether photic stimulation induced significant shifts in inter-cluster coupling strength (ΔR = Rlate − Rearly), a Two-Sample Circular Permutation Test (*N* = 2000 iterations) was executed for each FOV, with *p*-values adjusted for multiple comparisons using the Benjamini–Hochberg False Discovery Rate (FDR) procedure.

#### 4.4.6. Statistical Framework

Statistical analyses were performed using Python v3.14.8 (scipy.stats, statsmodels, pycircstat). Intra-cluster paired comparisons across temporal segments (e.g., early vs. late period) were conducted using the non-parametric Wilcoxon Signed-Rank Test. Inter-cluster comparisons for continuous features were performed using the omnibus Kruskal–Wallis test, followed by post-hoc Mann–Whitney U tests with Benjamini–Hochberg FDR correction for multiple hypothesis testing. Circular data were analyzed using non-parametric circular permutation routines. A threshold of *p_FDR_* < 0.05 was established for statistical significance across all tests.

## 5. Conclusions

In conclusion, our study demonstrates the utility of integrating continuous wavelet transform analysis with unsupervised clustering to decode the non-stationary intracellular Ca^2+^ dynamics of retinal neurons and Müller glial cells. By moving beyond static summary descriptors, this time-resolved framework uncovers distinct functional subpopulations defined by divergent kinetic regimes contrasting rapid light-evoked period transitions in developing neurons with multi-phasic kinetic trajectories and a decentralized mosaic of autonomy in macroglia. Furthermore, evaluating instantaneous phase coupling strictly within localized fields of view reveals stimulus-entrained temporal response sequencing across cell assemblies without overinterpreting causal network drive. Overall, this computational pipeline provides a robust, objective descriptive benchmark for quantifying functional heterogeneity and dynamic temporal coordination in retinal cell populations under photic stimulation.

## Figures and Tables

**Figure 1:**
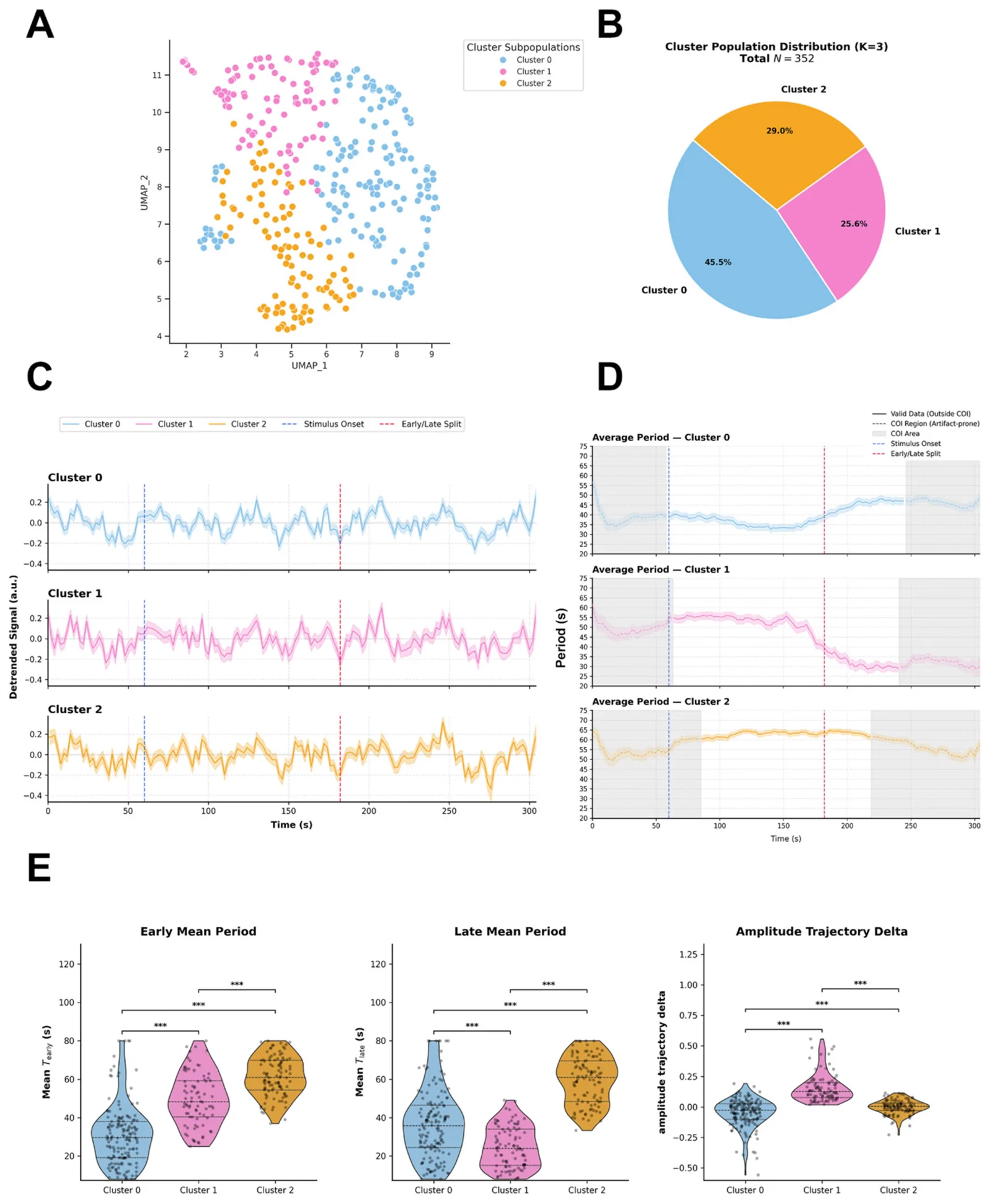
(**A**) Uniform Manifold Approximation and Projection (UMAP) embedding illustrates the two-dimensional distribution of mixed primary embryonic chicken retinal cells (*N* = 352 total cells). Color coding indicates subpopulation assignments across three optimal functional clusters (K = 3) derived from Agglomerative Hierarchical Clustering performed on the standardized high-dimensional feature space. (**B**) Pie chart depicting the numerical distribution and relative percentages of cells across the identified clusters: Cluster 0 (45.5%, *N* = 160), Cluster 1 (25.6%, *N* = 90), and Cluster 2 (29.0%, *N* = 102). (**C**) Mean detrended intracellular Ca^2+^ fluorescence traces (ΔF/F0, arbitrary units) over time for Cluster 0 (blue), Cluster 1 (pink), and Cluster 2 (orange). Vertical dashed blue lines mark blue light stimulus onset (T = 60 s) and vertical dashed red lines indicate the early/late segment boundary, shaded areas represent SEM. (**D**) Continuous instantaneous period curves (s) extracted across time for each cluster. Gray shaded boundaries denote the Cone of Influence (COI) estimated via pyBOAT, demarcating regions prone to spectral edge truncation artifacts. (**E**) Inter-Cluster Parametric Distributions (Violin Plots). Distribution of key kinetic and spectral features across clusters, Early Mean Period (Mean T_early), Late Mean Period (Mean T_late) and Amplitude Trajectory Delta. Non-parametric omnibus evaluations across clusters were conducted using Kruskal–Wallis H-tests. Pairwise inter-cluster comparisons were evaluated using post-hoc Mann–Whitney U tests with Benjamini–Hochberg false discovery rate (FDR) correction for multiple testing. Significance levels: *** *p* < 0.001. Data are displayed as violin density plots overlaid with individual data points, median horizontal lines, and interquartile ranges (IQR).

**Figure 2:**
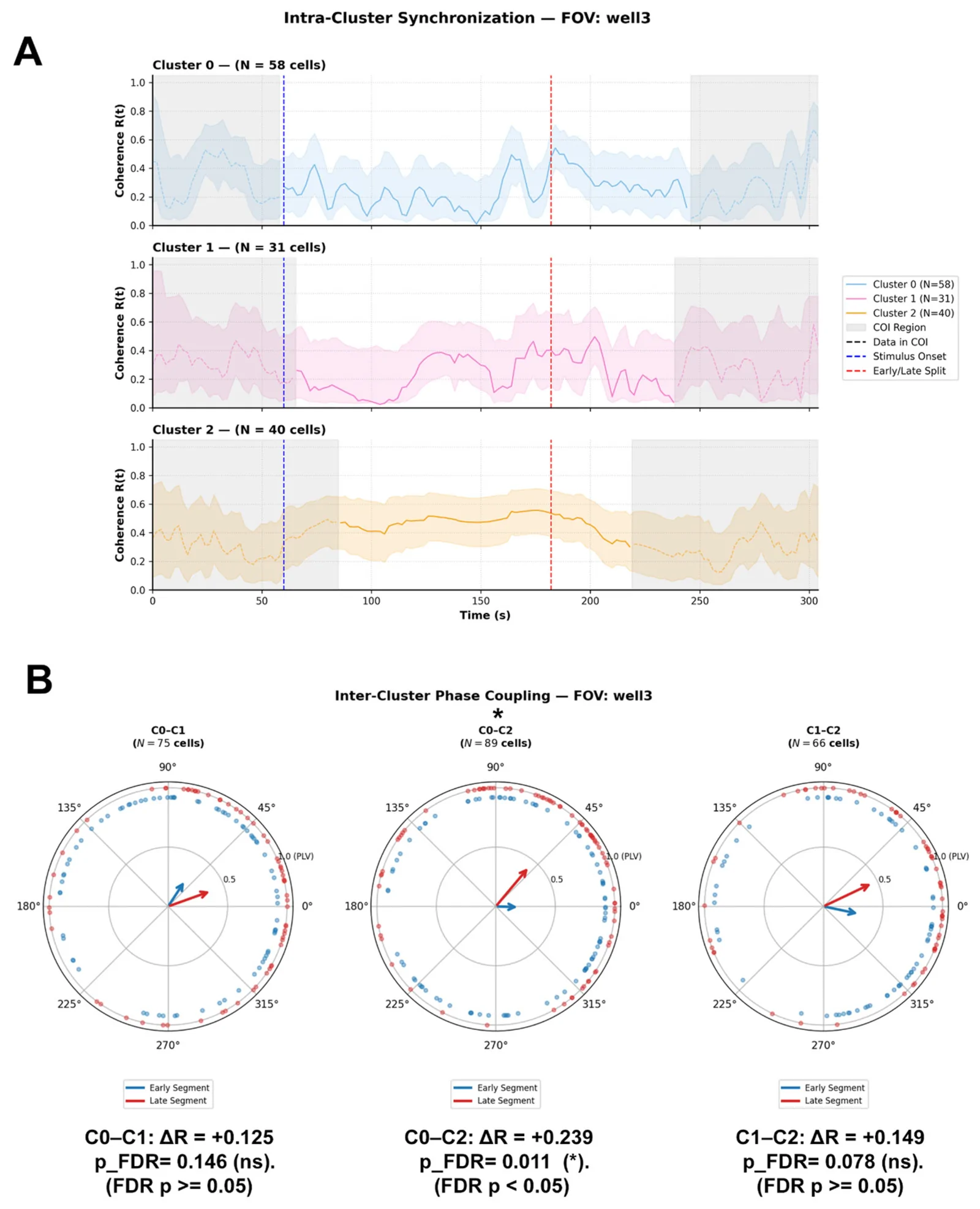
(**A**) Intra-Cluster Coherence (R(t)). Time-resolved mean phase coherence R(t) across cells within Cluster 0 (*N* = 58 cells, blue trace), Cluster 1 (*N* = 31 cells, pink trace), and Cluster 2 (*N* = 40 cells, orange trace) over a 300 s recording window. Vertical dashed blue lines indicate stimulus onset (T = 60), and vertical dashed red lines mark the boundary separating the early and late experimental segments. Shaded gray regions denote the Cone of Influence (COI), indicating boundary regions susceptible to spectral edge artifacts. Shaded color envelopes represent variability around the mean coherence. (**B**) Inter-Cluster Phase Coupling. Circular polar plots displaying phase difference distributions (ΔΦ) and Phase-Locking Value vectors (R = PLV) between cluster pairs: C0–C1 (*N* = 75 cells), C0–C2 (*N* = 89 cells), and C1–C2 (*N* = 66 cells). Blue vectors and markers illustrate phase coupling during the Early segment, whereas red vectors and markers represent the Late segment. Vector length reflects phase-locking strength (R), and vector direction indicates the mean phase difference. Effect size is expressed as the net difference in phase-locking value (Delta R = R_Late − R_Early). A statistically significant shift in phase coupling post-stimulus was observed exclusively between C0 and C2 (Delta R = +0.239, *p_FDR_* = 0.011, whereas shifts between C0–C1 (Delta R = +0.125, *p_FDR_* = 0.146 and C1–C2 (Delta R = +0.149, *p_FDR_* = 0.078) were non-significant. Statistical Analysis: Circular phase uniformity within each segment was evaluated using Rayleigh tests. Differences in phase coupling distributions between Early and Late segments were tested using a two-sample circular permutation test (*N* = 2000 iterations) with Benjamini–Hochberg False Discovery Rate (FDR) adjustment for multiple testing. Significance levels: * *p_FDR_* < 0.05.

**Figure 3:**
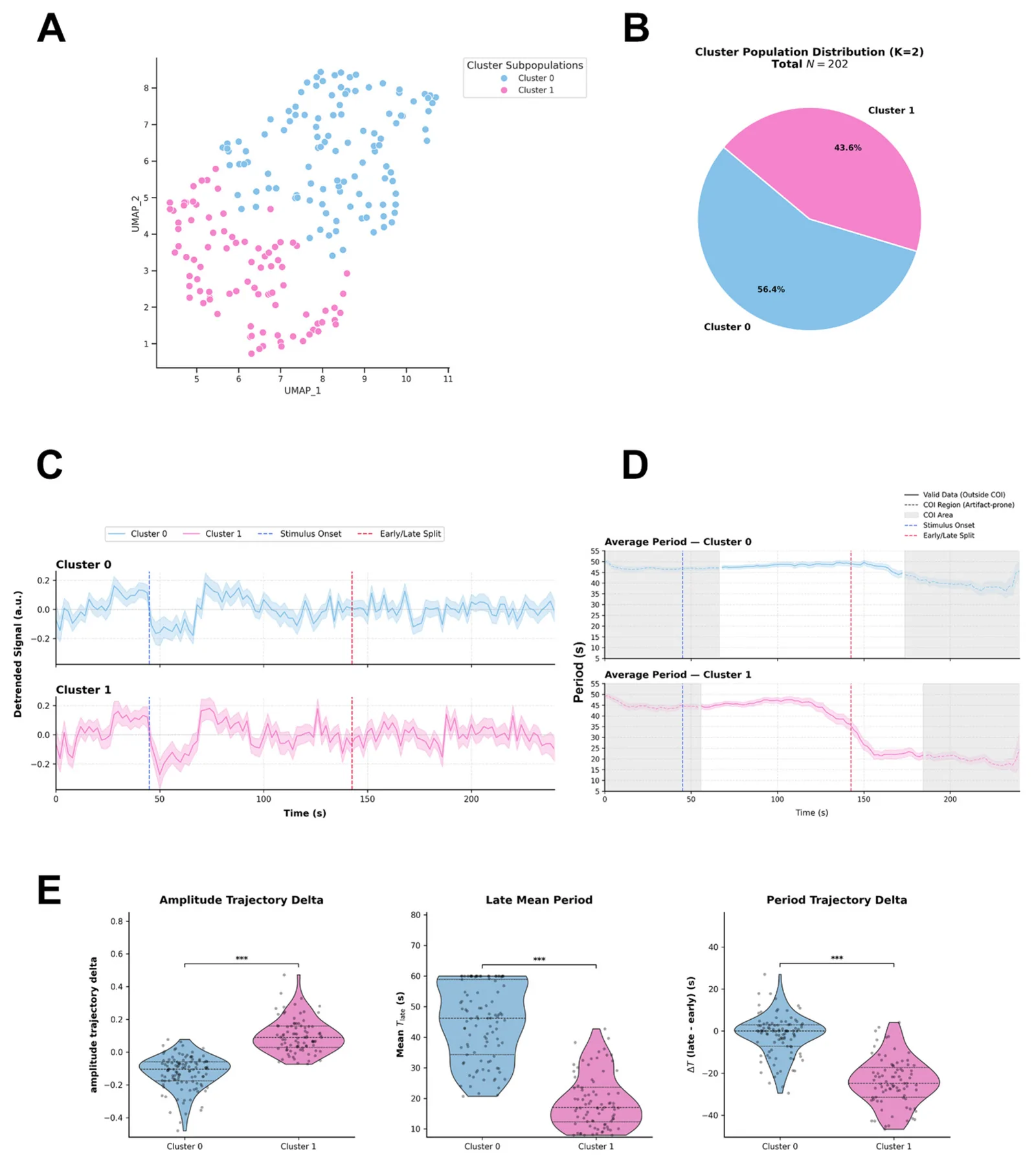
(**A**) Uniform Manifold Approximation and Projection (UMAP) embedding illustrates the two-dimensional distribution of chicken Müller Glial Cells (MGCs; *N* = 202 total cells). Color coding indicates subpopulation assignments across two optimal functional clusters (K = 2) derived from Agglomerative Hierarchical Clustering performed on the standardized high-dimensional feature space. (**B**) Pie chart depicting the numerical distribution and relative percentages of MGCs across the identified clusters: Cluster 0 (56.4%) and Cluster 1 (43.6%) for a total population of *N* = 202 cells. (**C**) Detrended Calcium Time-Series. Mean detrended intracellular Ca^2+^ fluorescence traces (ΔF/F0, arbitrary units) over time for Cluster 0 (blue) and Cluster 1 (pink). Vertical dashed blue lines mark stimulus onset and vertical dashed red lines indicate the early/late segment boundary. Shaded areas represent mean SEM. (**D**) Continuous instantaneous period curves (s) extracted across time for each cluster. Gray shaded boundaries denote the Cone of Influence (COI) estimated via pyBOAT, demarcating regions prone to spectral edge truncation artifacts. (**E**) Distribution of key kinetic and spectral features across MGC clusters: Amplitude Trajectory Delta, Late Mean Period (Mean T_late). Period Trajectory Delta (Delta T (late − early)). Statistical Analysis: Non-parametric omnibus evaluations across clusters were conducted using Kruskal–Wallis H-tests. Pairwise inter-cluster comparisons were evaluated using post-hoc Mann–Whitney U tests with Benjamini–Hochberg false discovery rate (FDR) correction for multiple testing. Significance levels: *** *p* < 0.001. Data are displayed as violin density plots overlaid with individual data points, median horizontal lines, and interquartile ranges (IQR).

**Figure 4:**
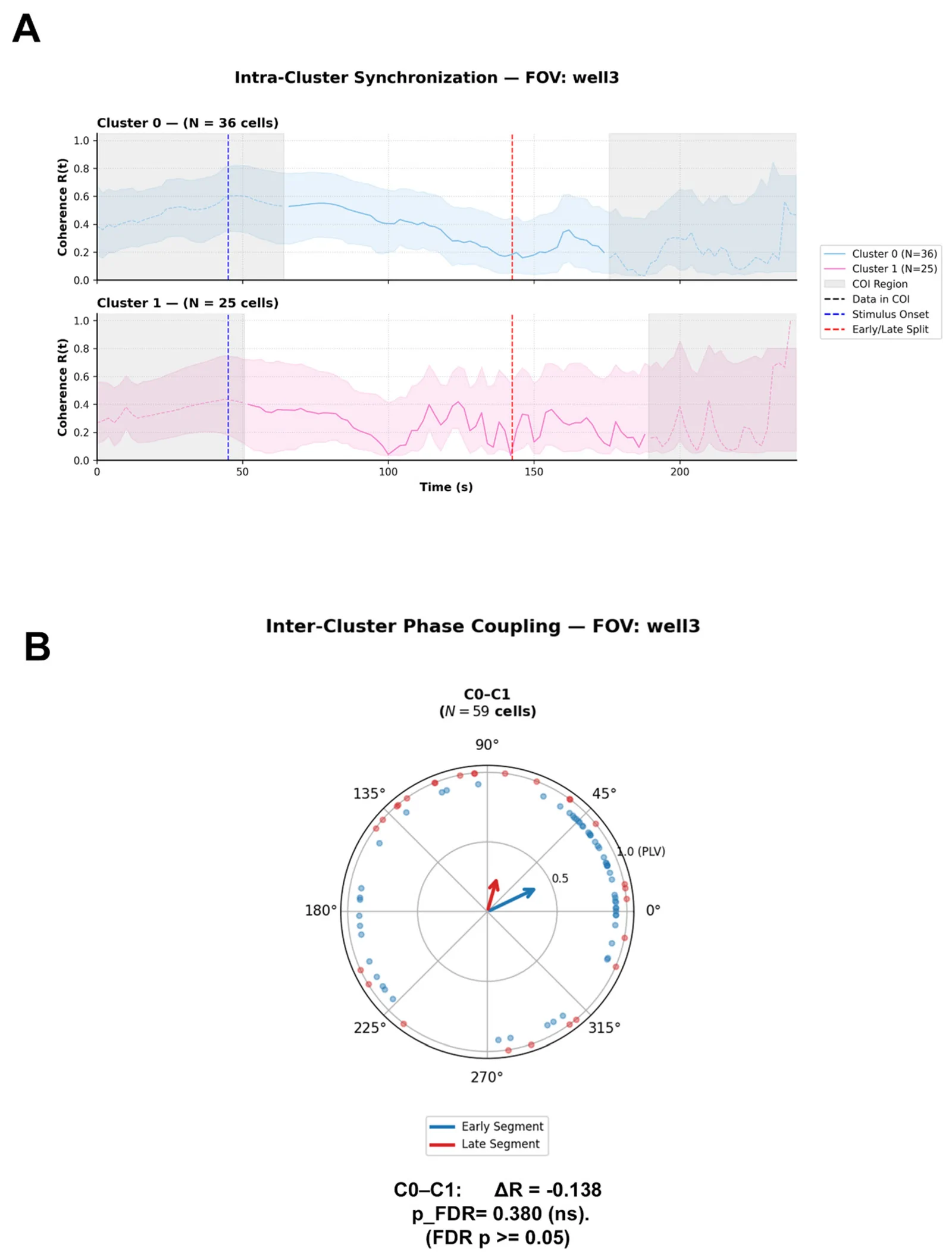
(**A**) Time-resolved mean phase coherence R(t) across cells within Cluster 0 (*N* = 36 cells, blue trace) and Cluster 1 (*N* = 25 cells, pink trace) over the recording window. Vertical dashed blue lines mark blue light stimulus onset (T = 45 s), and vertical dashed red lines indicate the boundary separating early and late experimental segments. Shaded gray regions denote the Cone of Influence (COI), indicating boundary regions susceptible to spectral edge truncation artifacts. Shaded color envelopes represent variability around the mean coherence. (**B**) Inter-Cluster Phase Coupling. Circular polar plot displaying phase difference distributions (ΔΦ) and Phase-Locking Value vectors (R = PLV) between the cluster pair C0–C1 (*N* = 59 cells). Blue vectors and markers illustrate phase coupling during the Early segment, whereas red vectors and markers represent the Late segment. Vector length reflects phase-locking strength (R), and vector direction indicates the mean phase difference. Effect size is expressed as the net difference in phase-locking value (Delta R = R_Late − R_Early). The shift in inter-cluster phase coupling post-stimulus was statistically non-significant (Delta R = -0.138, *p_FDR_* = 0.380). Statistical Analysis: Circular phase uniformity within each segment was evaluated using Rayleigh tests. Differences in phase coupling distributions between Early and Late segments were tested using a two-sample circular permutation test (*N* = 2000 iterations) with Benjamini–Hochberg False Discovery Rate (FDR) adjustment for multiple testing. Significance levels: ns = non-significant (*p_FDR_* > 0.05).

**Figure 5:**
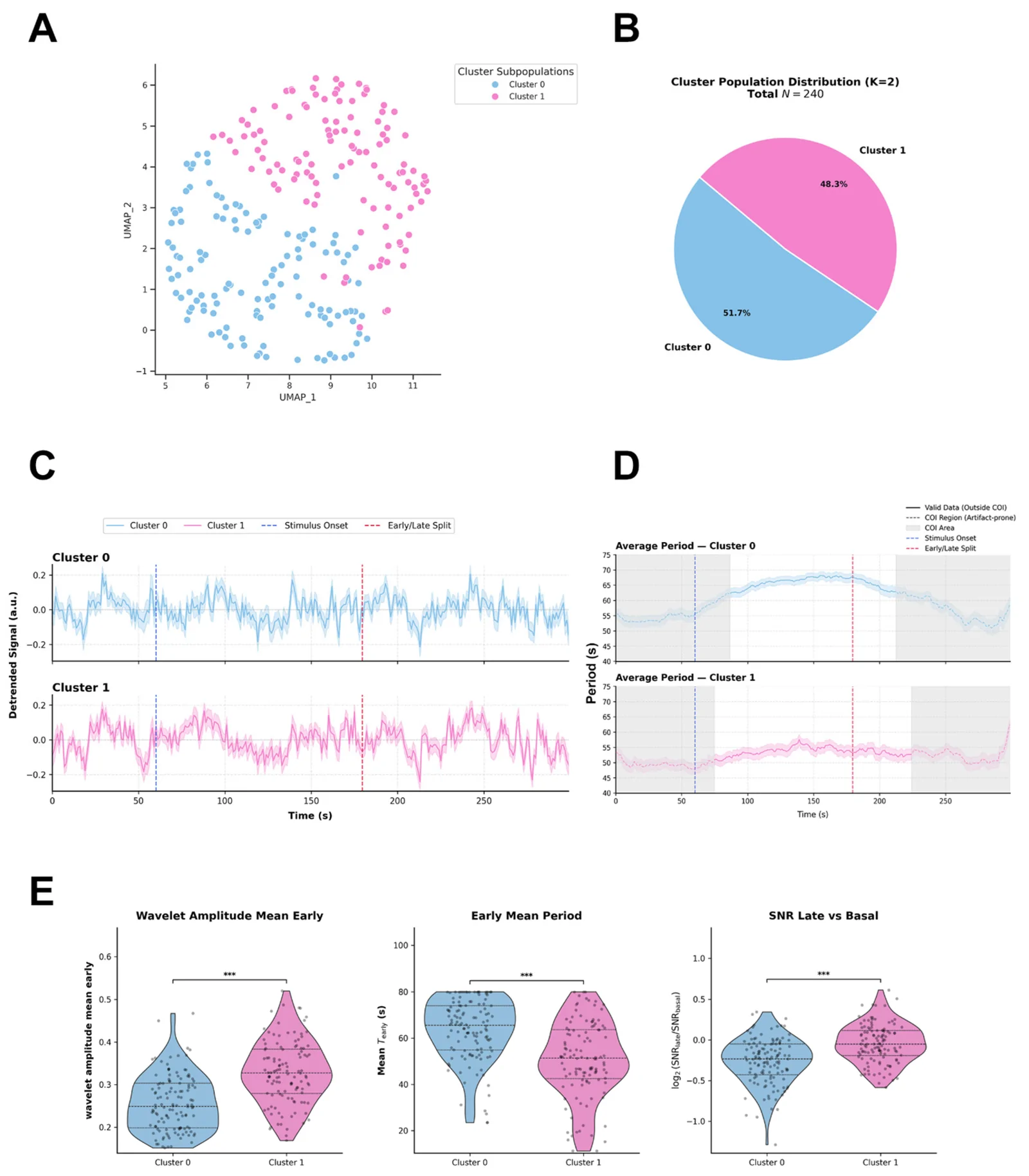
(**A**) Uniform Manifold Approximation and Projection (UMAP) embedding illustrates the two-dimensional distribution of human Müller glial cells (MIO-M1; *N* = 240 total cells). Color coding indicates subpopulation assignments across two optimal functional clusters (K = 2) derived from Agglomerative Hierarchical Clustering performed on the standardized high-dimensional feature space. (**B**) Pie chart depicting the numerical distribution and relative percentages of MIO-M1 cells across the identified clusters: Cluster 0 (51.7%) and Cluster 1 (48.3%) for a total population of *N* = 240 cells. (**C**) Mean detrended intracellular Ca^2+^ fluorescence traces (ΔF/F0, arbitrary units) over time for Cluster 0 (blue) and Cluster 1 (pink). Vertical dashed blue lines mark blue light stimulus onset (T = 60 s) and vertical dashed red lines indicate the early/late segment boundary. Shaded areas represent SEM. (**D**) Continuous instantaneous period curves (s) extracted across time for each cluster. Gray shaded boundaries denote the Cone of Influence (COI) estimated via pyBOAT, demarcating regions prone to spectral edge truncation artifacts. (**E**) Distribution of key kinetic and spectral features across MIO-M1 clusters: Wavelet Amplitude Mean Early, Early Mean Period (Mean T_early), and SNR (Signal-to-Noise Ratio) Late vs. Basal. Statistical Analysis: Non-parametric omnibus evaluations across clusters were conducted using Kruskal–Wallis H-tests. Pairwise inter-cluster comparisons were evaluated using post-hoc Mann–Whitney U tests with Benjamini–Hochberg false discovery rate (FDR) correction for multiple testing. Significance levels: *** *p* < 0.001. Data are displayed as violin density plots overlaid with individual data points, median horizontal lines, and interquartile ranges (IQR).

**Figure 6:**
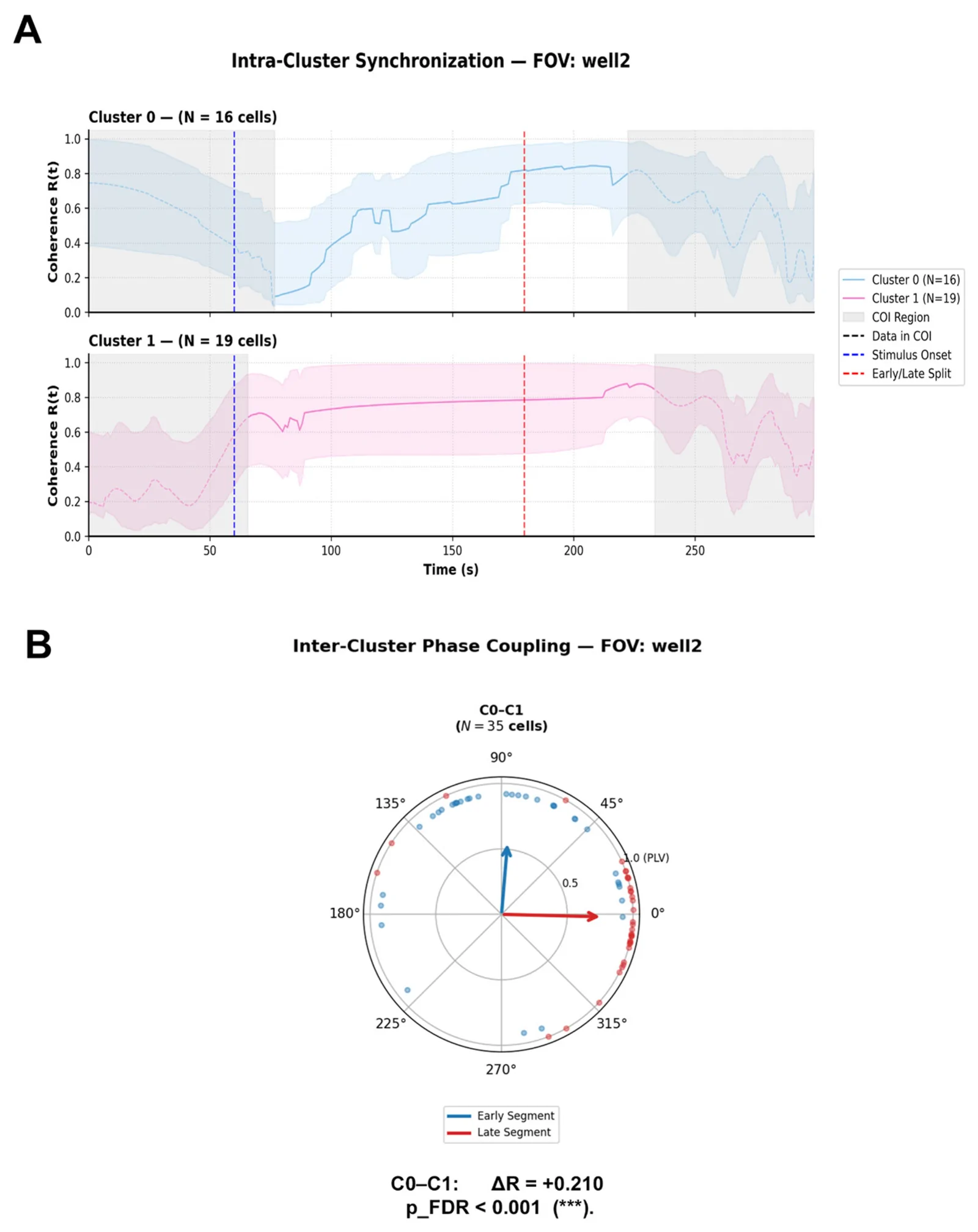
(**A**) Intra-Cluster Coherence (R(t)). Time-resolved mean phase coherence R(t) across cells within Cluster 0 (*N* = 16 cells, blue trace) and Cluster 1 (*N* = 19 cells, pink trace) over a 300 s recording window. Vertical dashed blue lines mark blue light stimulus onset (T = 60 s), and vertical dashed red lines indicate the boundary separating early and late experimental segments. Shaded gray regions denote the Cone of Influence (COI), indicating boundary regions susceptible to spectral edge truncation artifacts. Shaded color envelopes represent variability around the mean coherence. (**B**) Inter-Cluster Phase Coupling. Circular polar plot displaying phase difference distributions (ΔΦ) and Phase-Locking Value vectors (R = PLV) between the cluster pair C0–C1 (*N* = 35 cells). Blue vectors and markers illustrate phase coupling during the Early segment, whereas red vectors and markers represent the Late segment. Vector length reflects phase-locking strength (R), and vector direction indicates the mean phase difference. Effect size is expressed as the net difference in phase-locking value (Delta R = R_Late − R_Early). A statistically significant shift in inter-cluster phase coupling post-stimulus was observed between C0 and C1 (Delta R = +0.210, *p_FDR_* < 0.001). Statistical Analysis: Circular phase uniformity within each segment was evaluated using Rayleigh tests. Differences in phase coupling distributions between Early and Late segments were tested using a two-sample circular permutation test (*N* = 2000 iterations) with Benjamini–Hochberg False Discovery Rate (FDR) adjustment for multiple testing. Significance levels: *** *p_FDR_* < 0.001; *p_FDR_* < 0.01.

**Table 1:** Signal Processing and Time-Frequency Decomposition Parameters.

Parameter	MIO-M1	Neurons	MGCs Chicken
**Sampling Interval** (**Δt**)	1.0 s	2.0 s	2.0 s
**Period Range** (**PERIODS**)	6.0–80.0 s	8.0–80.0 s	8.0–60.0 s
**Period Resolution** (**Nevals**)	150 steps	150 steps	100 steps
**Sinc Cutoff Period** (**Tcutoff**)	100.0 s	100.0 s	75.0 s
**Envelope Window** (**Wenvelope**)	30.0 s	30.0 s	25.0 s
**Stimulus Onset** (**Tstim_start**)	60.0 s	60.0 s	45.0 s

## Data Availability

The datasets generated and analyzed during the current study, along with the processing scripts and the WAVE-CLUST model developed for this analysis, are publicly available in the GitHub repository at https://github.com/maxirios27/WAVE_CLUST.git (accessed on 1 May 2026).

## Supplementary Materials

The following supporting information can be downloaded at: https://www.mdpi.com/article/10.3390/ijms27198879/s1.

## Abbreviations

Ca^2+^	Calcium
CWT	Continuous Wavelet Transform
GCL	Ganglion Cell Layer
INL	Inner Nuclear Layer
RGCs	Retinal ganglion cells
MIO-M1	Moorfields/Institute of Ophthalmology-Müller 1
MGCs	Müller glial cells
ONL	Outer Nuclear Layer
Opn	Opsin
PLC	Phospholipase C
UMAP	Uniform Manifold Approximation and Projection
UV	Ultraviolet
COI	Cone of Influence
ROI	Region of Interest
SNR	Signal-to-Noise Ratio
FDR	False Discovery Rate
FOV	Field of View
DMEM	Dulbecco’s Modified Eagle Medium
PLV	Phase Locking Value
