# Supplementary material for: Unveiling Glial Heterogeneity in the Inner Retina: Non-Visual Photic Spectrum Responses and Calcium Dynamics in Müller Glial Cells vs. Neurons
Source: Int J Mol Sci. 2026 Oct 6;27(19):8879. doi: 10.3390/ijms27198879 (PMC13665424; doi:10.3390/ijms27198879)
Supplement: Supplementary file 1 [file ijms-27-08879-s001.zip › suplementary legens.pdf]

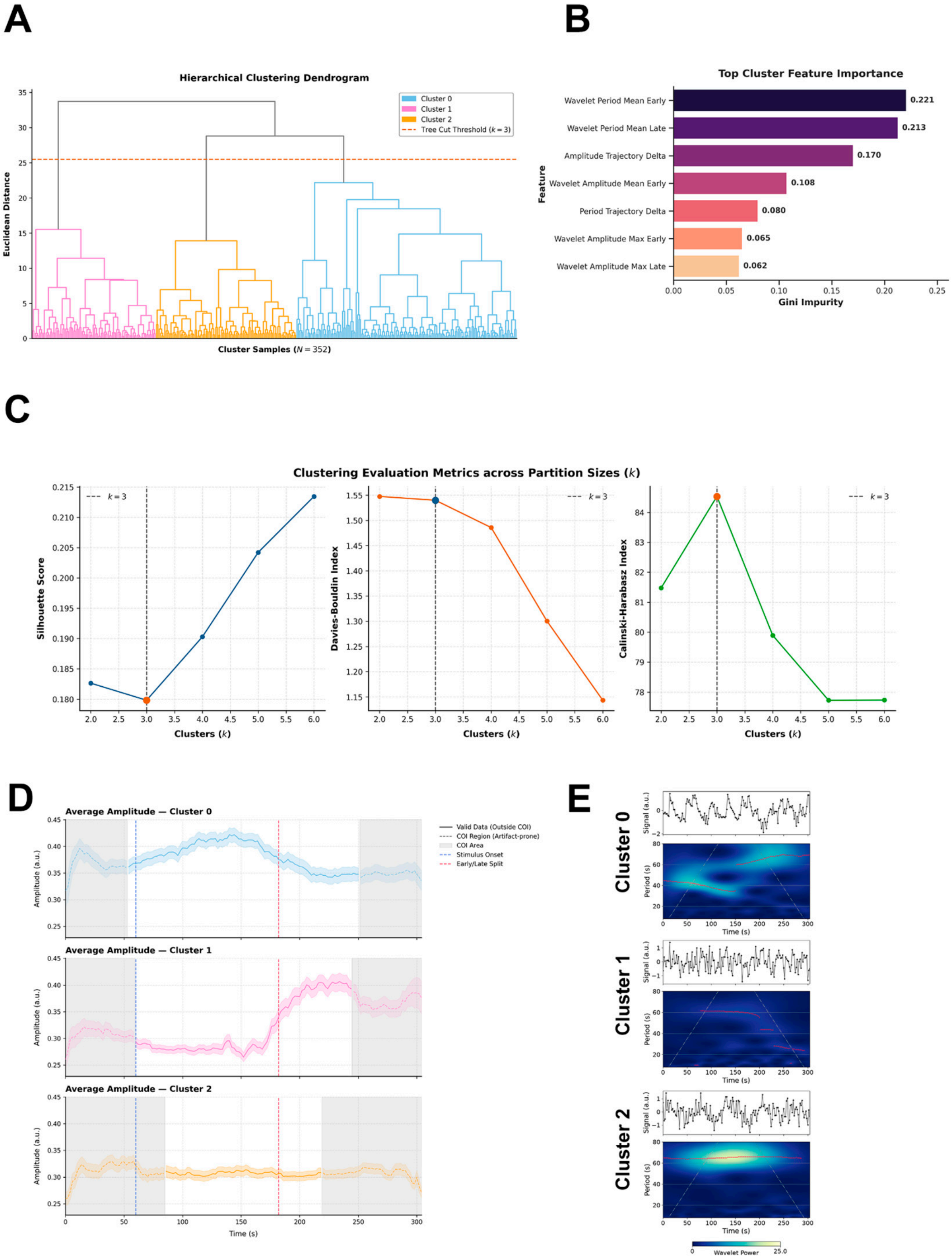

Supplementary Figure S1. Unsupervised hierarchical clustering validation, feature importance ranking, and wavelet spectral decomposition of primary embryonic retinal neurons.**(A)** Hierarchical Clustering Dendrogram. Agglomerative hierarchical clustering dendrogram based on Ward's linkage and Euclidean distance across standardized high-dimensional feature space (N = 352 total cells). The horizontal dashed line marks the tree cut threshold corresponding to the optimal three-cluster partition (k = 3), classifying cells into Cluster 0 (blue), Cluster 1 (pink), and Cluster 2 (orange).**(B)** Top Cluster Feature Importance. Bar plot ranking the top functional features by mean decrease in Gini impurity derived from Random Forest classification. The top 3 discriminative features driving subpopulation

partitioning include Wavelet Period Mean Early (0.221), Wavelet Period Mean Late (0.213) and Amplitude Trajectory Delta (0.170). **(C)** Clustering Evaluation Metrics across Partition Sizes (k). Internal clustering validation indices evaluated across partition numbers k = 2 through 6: Silhouette Score, Davies-Bouldin Index, and Calinski-Harabasz Index. Vertical dashed lines mark the chosen optimal partition at k = 3, which yields peak separation and structural cohesion. **(D)** Continuous Average Amplitude Trajectories. Time-resolved mean wavelet amplitude curves (a.u.) over the recording window for Cluster 0 (blue), Cluster 1 (pink), and Cluster 2 (orange). Vertical dashed blue lines indicate blue light stimulus onset (T = 60s), and vertical dashed red lines mark the early/late segment boundary. Gray shaded regions represent the Cone of Influence (COI) prone to spectral edge truncation artifacts. Shaded color envelopes denote mean with SEM. **(E)** Representative Single-Cell Calcium traces and wavelet power scalograms. Exemplar detrended intracellular Ca^{2+} fluorescence signals (Signal,) over time alongside their corresponding Continuous Wavelet Transform (CWT) power scalograms across instantaneous period scales for individual cells representative of Cluster 0, Cluster 1, and Cluster 2. Heatmap intensity corresponds to spectral wavelet power.

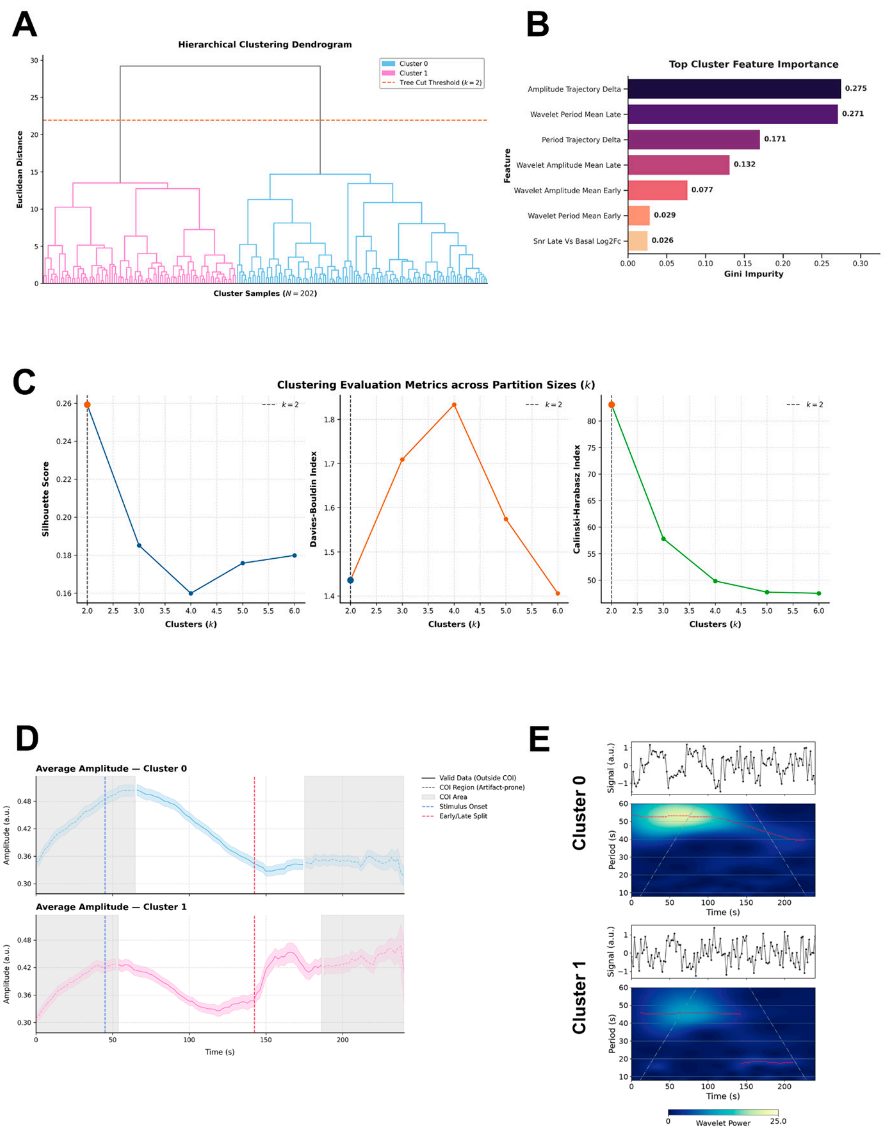

Supplementary Figure S2. Unsupervised hierarchical clustering validation, feature importance ranking, and wavelet spectral decomposition of chicken Müller Glial Cells (MGCs).**(A)** Hierarchical Clustering Dendrogram. Agglomerative hierarchical clustering dendrogram based on Ward's linkage

and Euclidean distance across standardized high-dimensional feature space (N = 202 total cells). The horizontal dashed line marks the tree cut threshold corresponding to the optimal two-cluster partition (k = 2), classifying MGCs into Cluster 0 (blue) and Cluster 1 (pink).**(B)** Top Cluster Feature Importance. Bar plot ranking the top functional features by mean decrease in Gini impurity derived from Random Forest classification. The top 3 discriminative features driving subpopulation partitioning include Amplitude Trajectory Delta (0.275), Wavelet Period Mean Late (0.271), Period Trajectory Delta (0.171). **(C)** Clustering Evaluation Metrics across Partition Sizes (k). Internal clustering validation indices evaluated across partition numbers k = 2 through 6: Silhouette Score, Davies-Bouldin Index, and Calinski-Harabasz Index. Vertical dashed lines mark the chosen optimal partition at k = 2, which yields peak separation and structural cohesion.**(D)** Continuous Average Amplitude Trajectories. Time-resolved mean wavelet amplitude curves (a.u.) over the recording window for Cluster 0 (blue) and Cluster 1 (pink). Vertical dashed blue lines indicate blue light stimulus onset (T = 45 s), and vertical dashed red lines mark the early/late segment boundary. Gray shaded regions represent the Cone of Influence (COI) prone to spectral edge truncation artifacts. Shaded color envelopes denote mean with SEM.**(E)** Representative Single-Cell Calcium Traces and Wavelet Power Scalograms. Exemplar detrended intracellular Ca^{2+} fluorescence signals (Signal, a.u.) over time alongside their corresponding Continuous Wavelet Transform (CWT) power scalograms across instantaneous period scales (s) for individual cells representative of Cluster 0 and Cluster 1. Heatmap intensity corresponds to spectral wavelet power.

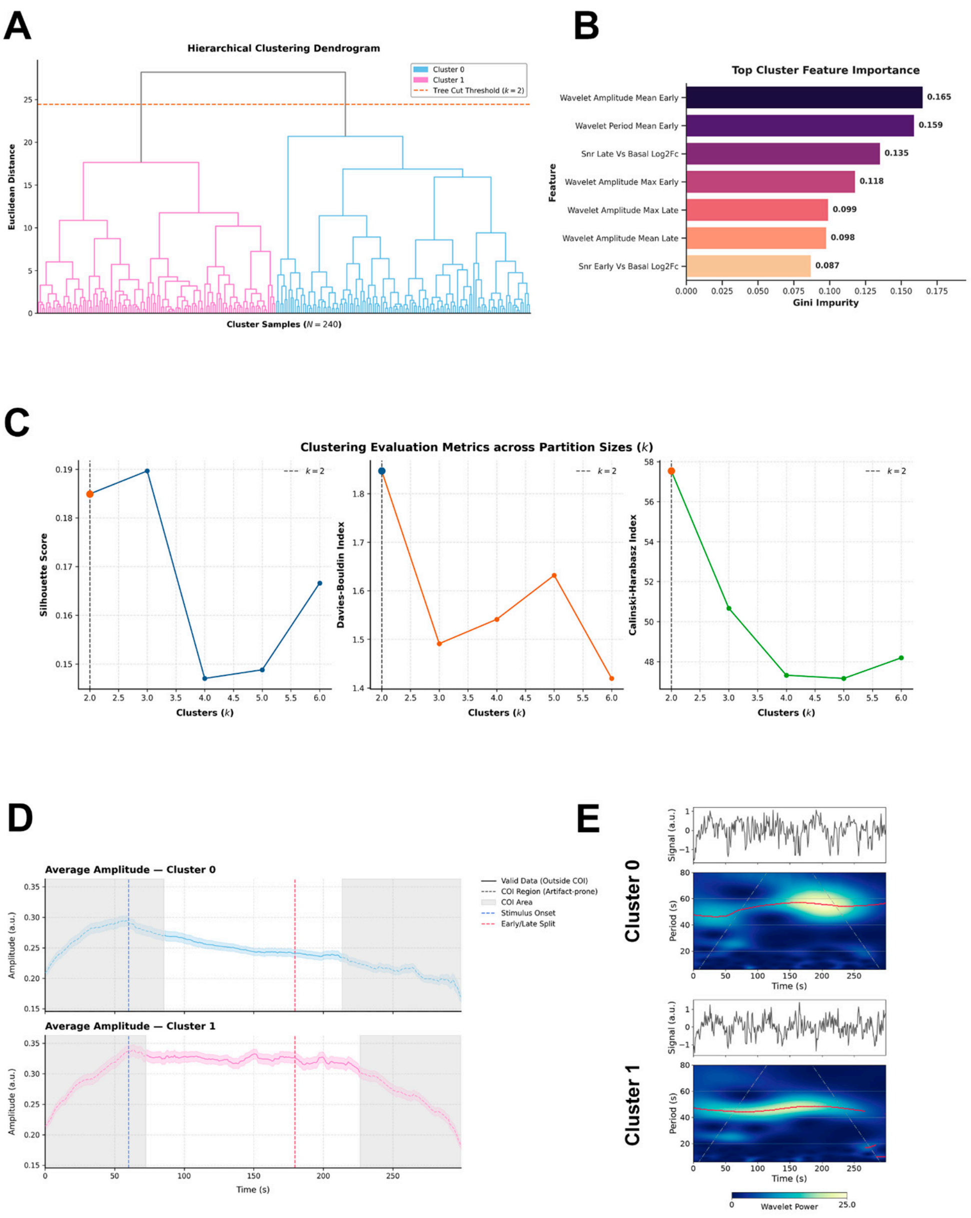

Supplementary Figure S3. Unsupervised hierarchical clustering validation, feature importance ranking, and wavelet spectral decomposition of human MIO-M1 cell line.**(A)** Hierarchical Clustering Dendrogram. Agglomerative hierarchical clustering dendrogram based on Ward's linkage and Euclidean distance across standardized high-dimensional feature space (N = 240 total cells). The horizontal dashed line marks the tree cut threshold corresponding to the optimal two-cluster partition (k = 2), classifying MGCs into Cluster 0 (blue) and Cluster 1 (pink).**(B)** Top Cluster Feature Importance. Bar plot ranking the top functional features by mean decrease in Gini impurity derived from Random Forest classification. The top 3 discriminative features driving subpopulation partitioning include Wavelet Amplitude mean Early (0.165), Wavelet Period Mean Early (0.159), SNR Late vs Basal log_2FC (0.135). **(C)** Clustering Evaluation Metrics across Partition Sizes (k). Internal clustering validation indices evaluated across partition numbers k = 2 through 6: Silhouette Score, Davies-Bouldin Index, and Calinski-Harabasz Index. Vertical dashed lines mark the chosen optimal partition at k = 2, which yields peak separation and structural cohesion. **(D)** Continuous Average Amplitude Trajectories. Time-resolved mean wavelet amplitude curves (a.u.) over the recording window for Cluster 0 (blue) and

Cluster 1 (pink). Vertical dashed blue lines indicate blue light stimulus onset (T = 45 s), and vertical dashed red lines mark the early/late segment boundary. Gray shaded regions represent the Cone of Influence (COI) prone to spectral edge truncation artifacts. Shaded color envelopes denote mean with SEM.**(E)** Representative Single-Cell Calcium Traces and Wavelet Power Scalograms. Exemplar detrended intracellular Ca^{2+} fluorescence signals (Signal, a.u.) over time alongside their corresponding Continuous Wavelet Transform (CWT) power scalograms across instantaneous period scales (s) for individual cells representative of Cluster 0 and Cluster 1. Heatmap intensity corresponds to spectral wavelet power.
