# Supplementary material for: Unveiling Glial Heterogeneity in the Inner Retina: Non-Visual Photic Spectrum Responses and Calcium Dynamics in Müller Glial Cells vs. Neurons
Source: Int J Mol Sci. 2026 Oct 6;27(19):8879. doi: 10.3390/ijms27198879 (PMC13665424; doi:10.3390/ijms27198879)
Supplement: Supplementary file 1 [file ijms-27-08879-s001.zip › Supplementary 4.pdf]

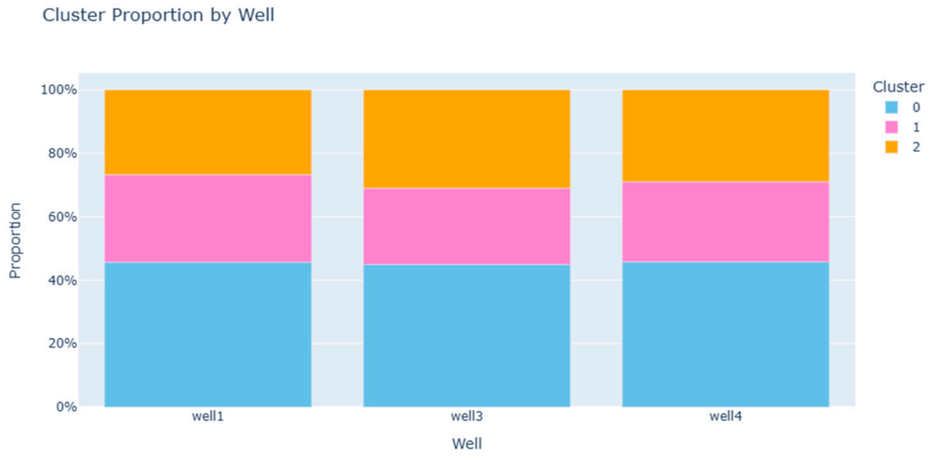

Neurons

--- Chi-squared Test for Independence of Clusters and Wells ---

Chi-squared Statistic: 0.71

P-value: 9.505e-01

Degrees of Freedom: 4

Conclusion: The P-value is greater than 0.05, suggesting no statistically significant association between the distribution of clusters and wells

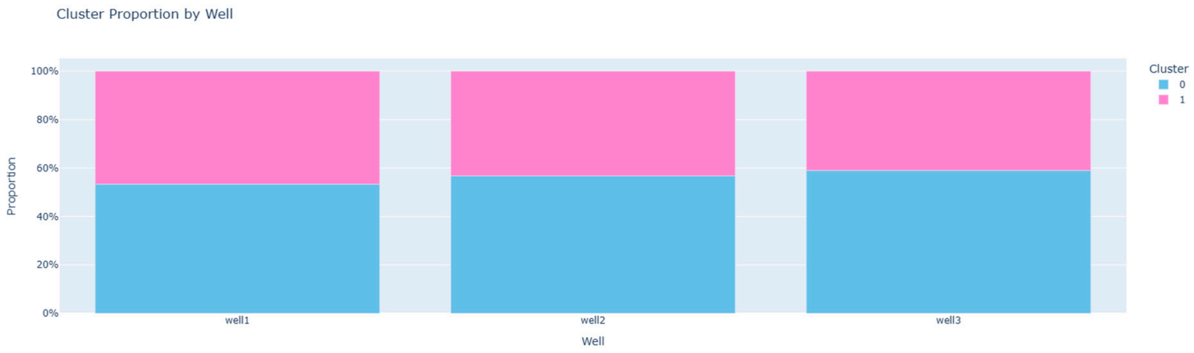

MGCs chicken

-- Chi-squared Test for Independence of Clusters and Wells ---

Chi-squared Statistic: 0.40

P-value: 8.170e-01

Degrees of Freedom: 2

Conclusion: The P-value is greater than 0.05, suggesting no statistically significant association between the distribution of clusters and wells

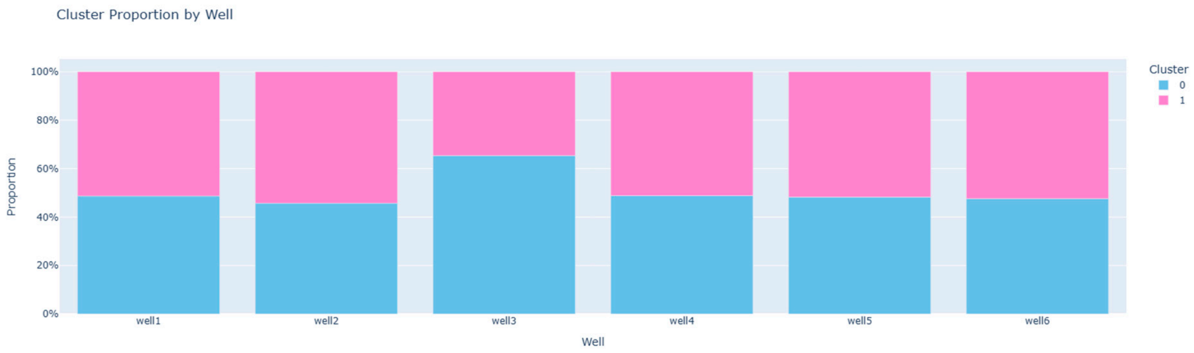

MIO-M1

-- Chi-squared Test for Independence of Clusters and Wells ---

Chi-squared Statistic: 5.10

P-value: 4.041e-01

Degrees of Freedom: 5

Conclusion: The P-value is greater than 0.05, suggesting no statistically significant association between the distribution of clusters and wells
