# Supplementary material for: Unveiling Glial Heterogeneity in the Inner Retina: Non-Visual Photic Spectrum Responses and Calcium Dynamics in Müller Glial Cells vs. Neurons
Source: Int J Mol Sci. 2026 Oct 6;27(19):8879. doi: 10.3390/ijms27198879 (PMC13665424; doi:10.3390/ijms27198879)
Supplement: Supplementary file 1 [file ijms-27-08879-s001.zip › Supplementary_Table_1_InterCluster_Phase_Coupling.pdf]

Supplementary Table 1. Comprehensive Statistical Analysis of Inter-Cluster Phase Coupling Dynamics Across Fields of View (FOVs)

Overview and Methodological Framework

- **Field-of-View Restraint:** Phase synchronization metrics were restricted strictly to cells recorded simultaneously within the same physical field of view (FOV / well) to prevent erroneous assumptions of physical coupling across independent preparations.
- **Analytical Window & COI Masking:** To eliminate Continuous Wavelet Transform (CWT) edge-truncation artifacts, analysis was confined to the safe analytical window estimated by PyBoat.
- **Evaluated Time Segments:** Statistical comparisons were conducted between the COI-safe early post-stimulus segment and the late post-stimulus recovery segment
- **Circular Statistical Testing:** 1. Rayleigh Test of Circular Uniformity: Applied per segment to confirm non-uniform distribution ($p < 0.05$) of instantaneous phase differences ($\Delta\varphi = \varphi_A - \varphi_B$) and extract mean phase angles (Mean $\Delta\varphi$).
  2. Two-Sample Circular Permutation Test: Performed with N = 2000 iterations to test differences in Phase Locking Value ($\Delta R = R_{late} - R_{early}$).
  3. Multiple Testing Correction: Benjamini-Hochberg False Discovery Rate (FDR) adjustment was applied across cluster pairs per FOV (q = 0.05).

FOV / Well	Cluster Pair	N (pairs)	Early Segment (Mean Δφ, R, p_Rayleigh)	Late Segment (Mean Δφ, R, p_Rayleigh)	ΔR (R_Late - R_Early)	p_raw (Permutation)	p_FDR (Benjamini-Hochberg)	Statistical Conclusion
well 1	C0–C1	69	+0.19 rad (+11.1°), R=0.409, $p=6.30\times10^{-6}$	-0.01 rad (-0.6°), R=0.317, $p=1.29\times10^{-3}$	-0.092	0.622	0.622	No significant shift (p_FDR ≥ 0.05)
well 1	C0–C2	74	+0.18 rad (+10.3°), R=0.313, $p=6.13\times10^{-4}$	-0.61 rad (-34.9°), R=0.399, $p=1.18\times10^{-5}$	+0.085	0.037	0.055	No significant shift (p_FDR ≥ 0.05)
well 1	C1–C2	57	+0.80 rad (+45.6°), R=0.444, $p=7.92\times10^{-6}$	-2.40 rad (-137.5°), R=0.303, $p=5.48\times10^{-3}$	-0.141	<0.001	<0.001	Significant shift, small effect size (||ΔR|| < 0.15)
well 3	C0–C1	75	+0.99 rad (+56.9°), R=0.264, $p=5.08\times10^{-3}$	+0.34 rad (+19.7°), R=0.388, $p=5.47\times10^{-5}$	+0.125	0.125	0.141	No significant shift (p_FDR ≥ 0.05)
**well 3**	**C0–C2**	**89**	**-0.03 rad (-1.8°), R=0.199, $p=2.89\times10^{-2}$**	**+0.87 rad (+50.1°), R=0.439, $p=1.26\times10^{-7}$**	**+0.239**	**0.005**	**0.011**	**Significant phase coupling shift (p_FDR < 0.05)**
well 3	C1–C2	66	-0.21 rad (-11.9°),	+0.44 rad (+25.5°),	+0.149	0.061	0.078	No significant

			R=0.311, $p=1.50\times10^{-3}$	R=0.460, $p=1.65\times10^{-6}$				shift (p_FDR ≥ 0.05)
**well 4**	**C0–C1**	**73**	**-0.41 rad (-23.3°), R=0.474, $p=3.85\times10^{-8}$**	**+0.21 rad (+12.0°), R=0.738, $p=4.22\times10^{-\pm7}$**	**+0.263**	**<0.001**	**<0.001**	**Significant phase coupling shift (p_FDR < 0.05)**
**well 4**	**C0–C2**	**77**	**-0.70 rad (-40.0°), R=0.343, $p=9.08\times10^{-5}$**	**-0.23 rad (-13.4°), R=0.743, $p=3.37\times10^{-i8}$**	**+0.399**	**<0.001**	**<0.001**	**Significant phase coupling shift (p_FDR < 0.05)**
**well 4**	**C1–C2**	**56**	**-0.76 rad (-43.3°), R=0.393, $p=1.57\times10^{-4}$**	**-0.31 rad (-17.8°), R=0.686, $p=5.45\times10^{-\pm2}$**	**+0.293**	**0.007**	**0.012**	**Significant phase coupling shift (p_FDR < 0.05)**

Footnotes & Metric Definitions

- **N (Cell Pairs):** Total number of valid functional cell pairs co-recorded within the indicated field of view fulfilling the sample size threshold ($N \geq 5$).
- **Mean Δφ:** Circular mean of instantaneous phase differences (φ_Cluster A - φ_Cluster B), expressed in radians and angular degrees.
- **R (Phase Locking Value / Vector Length):** Metric of phase coupling strength ranging from 0 (uniform distribution / lack of phase locking) to 1 (perfect phase locking).
- **p_Rayleigh:** Uniformity test p-value; $p < 0.05$ confirms a non-uniform phase distribution with a preferred relative phase angle.
- **ΔR (Effect Size):** Net change in phase locking strength between segments (ΔR = R_late - R_early). Positive values indicate photic reinforcement of inter-cluster phase coherence.
- **p_FDR:** Two-sample circular permutation test p-value adjusted for multiple comparisons via Benjamini-Hochberg FDR correction ($q = 0.05$). Significant shifts (p_FDR < 0.05) are highlighted in bold text.
