# Supplementary material for: Unveiling Glial Heterogeneity in the Inner Retina: Non-Visual Photic Spectrum Responses and Calcium Dynamics in Müller Glial Cells vs. Neurons
Source: Int J Mol Sci. 2026 Oct 6;27(19):8879. doi: 10.3390/ijms27198879 (PMC13665424; doi:10.3390/ijms27198879)
Supplement: Supplementary file 1 [file ijms-27-08879-s001.zip › Supplementary_Table_2_InterCluster_Phase_Coupling_MGCs.pdf]

Supplementary Table 2. Comprehensive Statistical Analysis of Inter-Cluster Phase Coupling Dynamics Across Fields of View (FOVs)

Overview and Methodological Framework

- **Field-of-View Restraint:** Phase synchronization metrics were restricted strictly to cells recorded simultaneously within the same physical field of view (FOV / well) to prevent erroneous assumptions of physical coupling across independent preparations.
- **Analytical Window & COI Masking:** To eliminate Continuous Wavelet Transform (CWT) edge-truncation artifacts, analysis was confined to the safe analytical window estimated by PyBoat.
- **Evaluated Time Segments:** Statistical comparisons were conducted between the COI-safe early post-stimulus segment and the late post-stimulus recovery segment.
- **Circular Statistical Testing:** 1. Rayleigh Test of Circular Uniformity: Applied per segment to confirm non-uniform distribution ($p < 0.05$) of instantaneous phase differences ($\Delta\varphi = \varphi\_A - \varphi\_B$) and extract mean phase angles (Mean $\Delta\varphi$).
  2. Two-Sample Circular Permutation Test: Performed with N = 2000 iterations to test differences in Phase Locking Value ($\Delta R = R\_late - R\_early$).
  3. Multiple Testing Correction: Benjamini-Hochberg False Discovery Rate (FDR) adjustment was applied across cluster pairs per FOV (q = 0.05).

FOV / Well	Cluster Pair	N (pairs)	Early Segment (Mean Δφ, R, p_Rayleigh)	Late Segment (Mean Δφ, R, p_Rayleigh)	ΔR (R_Late - R_Early)	p_raw (Permutation)	p_FDR (Benjamini-Hochberg)	Statistical Conclusion
well 1	C0–C1	59	+2.88 rad (+165.1°), R=0.134, p=0.350	+0.31 rad (+17.9°), R=0.153, p=0.661	+0.020	0.337	0.505	No significant shift (p_FDR ≥ 0.05)
well 2	C0–C1	78	+1.06 rad (+60.6°), R=0.149, p=0.179	+1.51 rad (+86.4°), R=0.041, p=0.942	-0.108	0.719	0.719	No significant shift (p_FDR ≥ 0.05)
well 3	C0–C1	59	+0.45 rad (+25.6°), R=0.416, $p=2.51\times10^{-5}$	+1.29 rad (+74.2°), R=0.278, p=0.124	-0.138	0.126	0.378	No significant shift (p_FDR ≥ 0.05)

Footnotes & Metric Definitions

- **N (Cell Pairs):** Total number of valid functional cell pairs co-recorded within the indicated field of view fulfilling the sample size threshold (N ≥ 5).
- **Mean Δφ:** Circular mean of instantaneous phase differences ($\varphi\_Cluster A - \varphi\_Cluster B$), expressed in radians and angular degrees.
- **R (Phase Locking Value / Vector Length):** Metric of phase coupling strength ranging from 0 (uniform distribution / lack of phase locking) to 1 (perfect phase locking).
- **p_Rayleigh:** Uniformity test p-value; $p < 0.05$ confirms a non-uniform phase distribution with a preferred relative phase angle.
- **ΔR (Effect Size):** Net change in phase locking strength between segments ($\Delta R = R\_late - R\_early$). Positive values indicate photic reinforcement of inter-cluster phase coherence.

- **p_FDR:** Two-sample circular permutation test p-value adjusted for multiple comparisons via Benjamini-Hochberg FDR correction ($q = 0.05$). Significant shifts ($p\_FDR < 0.05$) are highlighted in bold text.
