# Supplementary material for: Unveiling Glial Heterogeneity in the Inner Retina: Non-Visual Photic Spectrum Responses and Calcium Dynamics in Müller Glial Cells vs. Neurons
Source: Int J Mol Sci. 2026 Oct 6;27(19):8879. doi: 10.3390/ijms27198879 (PMC13665424; doi:10.3390/ijms27198879)
Supplement: Supplementary file 1 [file ijms-27-08879-s001.zip › Supplementary_Table_3_InterCluster_Phase_Coupling_MIO_M1.pdf]

Supplementary Table 3. Comprehensive Statistical Analysis of Inter-Cluster Phase Coupling Dynamics Across Fields of View (FOVs)

Overview and Methodological Framework

- **Field-of-View Restraint:** Phase synchronization metrics were restricted strictly to cells recorded simultaneously within the same physical field of view (FOV / well) to prevent erroneous assumptions of physical coupling across independent preparations.
- **Analytical Window & COI Masking:** To eliminate Continuous Wavelet Transform (CWT) edge-truncation artifacts, analysis was confined to the safe analytical window estimated by PyBoat.
- **Evaluated Time Segments:** Statistical comparisons were conducted between the COI-safe early post-stimulus segment and the late post-stimulus recovery segment.
- **Circular Statistical Testing:** 1. Rayleigh Test of Circular Uniformity: Applied per segment to confirm non-uniform distribution ($p < 0.05$) of instantaneous phase differences ($\Delta\varphi = \varphi\_A - \varphi\_B$) and extract mean phase angles (Mean $\Delta\varphi$).
  2. Two-Sample Circular Permutation Test: Performed with N = 2000 iterations to test differences in Phase Locking Value ($\Delta R = R\_late - R\_early$).
  3. Multiple Testing Correction: Benjamini-Hochberg False Discovery Rate (FDR) adjustment was applied across cluster pairs per FOV ($q = 0.05$).

FOV / Well	Cluster Pair	N (pairs)	Early Segment (Mean Δφ, R, p_Rayleigh)	Late Segment (Mean Δφ, R, p_Rayleigh)	ΔR (R_Late - R_Early)	p_raw (Permutation)	p_FDR (Benjamini-Hochberg)	Statistical Conclusion
well 1	C0–C1	39	-2.77 rad (-159.0°), R=0.242, p=0.102	+1.08 rad (+62.0°), R=0.283, p=0.047	+0.041	0.007	0.020	**Significant shift, small effect size (|ΔR| < 0.15)**
well 2	C0–C1	35	+1.49 rad (+85.2°), R=0.560, $p=7.30\times10^{-6}$	-0.02 rad (-1.4°), R=0.770, $p=1.14\times10^{-9}$	+0.210	<0.001	<0.001	**Significant phase coupling shift (p_FDR < 0.05)**
well 3	C0–C1	52	+0.11 rad (+6.5°), R=0.508, $p=6.39\times10^{-7}$	-0.20 rad (-11.4°), R=0.432, $p=4.05\times10^{-5}$	-0.076	0.370	0.444	No significant shift (p_FDR ≥ 0.05)
well 4	C0–C1	43	-2.24 rad (-128.6°), R=0.282, p=0.032	-1.00 rad (-57.3°), R=0.392, $p=1.11\times10^{-3}$	+0.110	0.040	0.080	No significant shift (p_FDR ≥ 0.05)
well 5	C0–C1	29	-0.44 rad (-25.4°), R=0.261, p=0.138	-2.38 rad (-136.3°), R=0.226, p=0.229	-0.035	0.090	0.134	No significant shift (p_FDR ≥ 0.05)
well 6	C0–C1	42	-0.62 rad (-35.5°), R=0.444, $p=1.80\times10^{-4}$	-0.45 rad (-25.5°), R=0.404, $p=8.52\times10^{-4}$	-0.040	0.831	0.831	No significant shift (p_FDR ≥ 0.05)

Footnotes & Metric Definitions

- **N (Cell Pairs):** Total number of valid functional cell pairs co-recorded within the indicated field of view fulfilling the sample size threshold ($N \geq 5$).
- **Mean Δφ:** Circular mean of instantaneous phase differences ($\varphi_{Cluster A} - \varphi_{Cluster B}$), expressed in radians and angular degrees.
- **R (Phase Locking Value / Vector Length):** Metric of phase coupling strength ranging from 0 (uniform distribution / lack of phase locking) to 1 (perfect phase locking).
- **p_Rayleigh:** Uniformity test p-value; $p < 0.05$ confirms a non-uniform phase distribution with a preferred relative phase angle.
- **ΔR (Effect Size):** Net change in phase locking strength between segments ($\Delta R = R_{late} - R_{early}$). Positive values indicate photic reinforcement of inter-cluster phase coherence.
- **p_FDR:** Two-sample circular permutation test p-value adjusted for multiple comparisons via Benjamini-Hochberg FDR correction ($q = 0.05$). Significant shifts ($p_{FDR} < 0.05$) are highlighted in bold text.
